# Macroporous Granular Hydrogels Functionalized with Aligned Architecture and Small Extracellular Vesicles Stimulate Osteoporotic Tendon‐To‐Bone Healing

**DOI:** 10.1002/advs.202304090

**Published:** 2023-10-22

**Authors:** Wei Song, Zhijie Ma, Xin Wang, Yifei Wang, Di Wu, Chongyang Wang, Dan He, Lingzhi Kong, Weilin Yu, Jiao Jiao Li, Haiyan Li, Yaohua He

**Affiliations:** ^1^ Department of Orthopedic Surgery Shanghai Sixth People's Hospital Affiliated to Shanghai Jiao Tong University School of Medicine Shanghai 200233 China; ^2^ School of Biomedical Engineering Shanghai Jiao Tong University Shanghai 200030 China; ^3^ School of Biomedical Engineering Faculty of Engineering and IT University of Technology Sydney Sydney New South Wales 2007 Australia; ^4^ Chemical and Environmental Engineering Department School of Engineering STEM College RMIT University 124 La Trobe St. Melbourne Victoria 3000 Australia; ^5^ Department of Orthopedic Surgery Jinshan District Central Hospital affiliated to Shanghai University of Medicine & Health Sciences Jinshan Branch of Shanghai Sixth People's Hospital Shanghai 201500 China

**Keywords:** aligned architecture, immunomodulation, macroporous granular hydrogels, osteoporotic tendon‐to‐bone healing, small extracellular vesicles

## Abstract

Osteoporotic tendon‐to‐bone healing (TBH) after rotator cuff repair (RCR) is a significant orthopedic challenge. Considering the aligned architecture of the tendon, inflammatory microenvironment at the injury site, and the need for endogenous cell/tissue infiltration, there is an imminent need for an ideal scaffold to promote TBH that has aligned architecture, ability to modulate inflammation, and macroporous structure. Herein, a novel macroporous hydrogel comprising sodium alginate/hyaluronic acid/small extracellular vesicles from adipose‐derived stem cells (sEVs) (MHA‐sEVs) with aligned architecture and immunomodulatory ability is fabricated. When implanted subcutaneously, MHA‐sEVs significantly improve cell infiltration and tissue integration through its macroporous structure. When applied to the osteoporotic RCR model, MHA‐sEVs promote TBH by improving tendon repair through macroporous aligned architecture while enhancing bone regeneration by modulating inflammation. Notably, the biomechanical strength of MHA‐sEVs is approximately two times higher than the control group, indicating great potential in reducing postoperative retear rates. Further cell‐hydrogel interaction studies reveal that the alignment of microfiber gels in MHA‐sEVs induces tenogenic differentiation of tendon‐derived stem cells, while sEVs improve mitochondrial dysfunction in M1 macrophages (Mφ) and inhibit Mφ polarization toward M1 via nuclear factor‐kappaB (NF‐κb) signaling pathway. Taken together, MHA‐sEVs provide a promising strategy for future clinical application in promoting osteoporotic TBH.

## Graphical abstract

A novel macroporous granular hydrogel comprising sodium alginate/hyaluronic acid/small extracellular vesicles from adipose‐derived stem cells (sEVs) (MHA‐sEVs) with aligned architecture and immunomodulatory properties is fabricated. When applied to the osteoporotic rotator cuff repair model, MHA‐sEVs promotes tendon‐to‐bone healing by improving tendon repair through macroporous aligned architecture while enhancing bone regeneration by modulating inflammation.

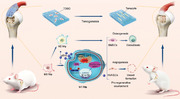

## 1 Introduction

Rotator cuff injury is one of the most common musculoskeletal disorders, and its incidence increases with age. Massive rotator cuff tears affect 25% of people over the age of 60% and 50% of people over the age of 80.^[^ ^1^ ^]^ It is estimated that ≈250 000 rotator cuff repair (RCR) surgeries are performed each year in the United States at a cost of nearly $3 billion.^[^ ^2^ ^]^ Despite advances in surgical techniques for RCR, 26−40% of repairs still experience structural failures.^[^ ^3^ ^]^ Current research on RCR attempts to satisfy a prevailing need to promote tendon‐to‐bone healing (TBH) and decrease postoperative retear rates.

Supraspinatus tendon quality is an important determinant of TBH following rotator cuff reconstruction.^[^ ^4^ ^]^ Normal supraspinatus tendon exhibits diversely aligned collagen fibers, with collagen type I (COL I) oriented parallel to the mechanical axis. The orientation of collagen fibers contributes to high tendon strength in the direction of fiber alignment. However, the tendon undergoes intrinsic degeneration following a rotator cuff tear. Extracellular matrix (ECM) produced by tenocytes decreases, and the density of collagen type III (COL III) in ECM increases, resulting in collagen degradation and fiber disorientation.^[^ ^5^ ^]^ Impaired tendon quality is the main reason leading to rotator cuff retear due to reduced resistance to external forces.^[^ ^6^ ^]^ Additionally, despite a significantly higher rate of revision surgery in patients with osteoporosis who underwent arthroscopic RCR, osteoporosis is often overlooked in the treatment of rotator cuff injuries.^[^ ^7^ ^]^ Osteoporosis can significantly affect the integrity of TBH following arthroscopic RCR due to its negative effects on bone quality.^[^ ^8^ ^]^ On one hand, it hinders tendon‐to‐bone interface reconstruction and predisposes the repaired tendon to anchor loosening and even pull‐out before TBH is achieved.^[^ ^9^ ^]^ On the other hand, rotator cuff injuries can independently lead to osteoporosis in the affected limb, especially the proximal humerus, due to loss of mechanical stimulation.^[^ ^10^ ^]^ These two conditions are frequently co‐present since a significant proportion of patients with rotator cuff injuries are from an aging population, particularly postmenopausal women who already suffer from osteoporosis. Taken together, to help TBH in the presence of osteoporosis, it is critical to simultaneously improve the quality of the regenerated supraspinatus tendon and bone regeneration.

Fiber scaffolds with an aligned structure have been shown to improve the quality of tendon regeneration in tissue engineering by promoting tenogenic differentiation of stem cells in the absence of growth factors, as well as ECM synthesis that chemically and structurally mimics the aligned structure of the tendon.^[^ ^11^ ^]^ Previously, the majority of fiber scaffolds applied for tendon regeneration were fabricated by electrospinning. However, the process is not cell/biomolecule‐friendly, and cell penetration is challenging due to the small pores of electrospun fibrous scaffolds.^[^ ^12^ ^]^ Hydrogels have been used for RCR due to their good biocompatibility and ability to mimic tendon ECM. However, the existing hydrogels usually have a nanoporous structure that physically constrains cells, significantly limiting migration and infiltration of cells/tissues and impairing the transportation of nutrients, molecules, and metabolites.^[^ ^13^ ^]^ Recently, macroporous granular hydrogels with pore sizes ranging from several to hundreds of microns have emerged as a new type of hydrogel for tackling the problems of traditional nanoporous hydrogels (NH), as they can better facilitate nutrient exchange and improve cell adhesion, proliferation, penetration, and ECM deposition.^[^ ^14^ ^]^ For example, our recent study synthesized sodium alginate (SA)/hyaluronic acid (HA) macroporous granular hydrogels by assembling SA/HA microfiber gels, which were shown to improve cell/tissue infiltration into the hydrogel and enhance cartilage regeneration in rat knee osteochondral defects.^[^ ^15^ ^]^ Although macroporous hydrogels have demonstrated improved functionality in several types of tissue regeneration, they have never been used for TBH, mainly due to the limited methods for fabricating macroporous hydrogels with aligned architecture.^[^ ^12^, ^13^, ^16^ ^]^

Few methods are available for preparing highly ordered macroporous hydrogels, including the template method, the fragmentation method of pressing nanoporous hydrogels through a grid, and the method of collecting high‐aspect‐ratio microgels with a rotating collector.^[^ ^12^, ^17^ ^]^ For example, micrometer‐sized sodium acetate (NaAc)·3H_2_O crystal templates with aligned microstructures were formed by crystallization from a supersaturated NaAc solution.^[^ ^17a^ ^]^ An agarose hydrogel with aligned microstructure and macropores was obtained by removing these micrometer‐sized aligned crystal templates from the hydrogel. However, the temperature reached 90 °C during preparation, which excluded the delivery of bioactive substances such as stem cells or growth factors. Others have pressed a pre‐cross‐linked bulk hydrogel through a grid with an adjustable opening size to form macroporous‐aligned hydrogel microstrands.^[^ ^17c^ ^]^ Despite the simplicity of this method, it required pre‐crosslinking of the hydrogel and also posed difficulties for stable hydrogels to pass through small grid pores. Additionally, preparing hydrogels suitable for rotator cuff implantation imposes high technical requirements on the manufacture of nylon grids. A wet spinning technique has been used to fabricate microgels with high‐aspect‐ratio, such as microribbons and microfiber gels, which can be assembled into macroporous hydrogels.^[^ ^18^ ^]^ One study used a rotating magnet‐containing collector to align high‐aspect‐ratio gelatin‐based microribbons for producing macroporous hydrogels.^[^ ^12^ ^]^ However, this alignment method required post‐treatments on the microribbons with ethanol and methanol, which would destroy any loaded bioactive substances. Taken together, existing methods of preparing macroporous hydrogels with aligned architecture usually involve harsh fabrication conditions, additional chemicals, and specific equipment, making them impractical for clinical application. Thus, there is high demand for an effective and practical method to produce macroporous hydrogels through aligned microgels that can be applied in an operation room.

In addition to providing physical cues through an aligned architecture, scaffolds for TBH should also supply biochemical cues, especially for bone regeneration. The inflammatory microenvironment medicated by M1 macrophages (Mφ) due to mitochondrial dysfunction plays a crucial role in osteoporotic bone regeneration.^[^ ^19^ ^]^ It has been reported that modulating Mφ polarization to the M2 phenotype in the early stage of TBH can inhibit their secretion of excessive inflammatory factors, stimulating vascularization and bone regeneration.^[^ ^20^ ^]^ Therefore, improving M1 Mφ mitochondrial function and inhibiting M0 Mφ polarization to M1 is expected to enhance bone regeneration after RCR and create favorable conditions for TBH. Small extracellular vesicles derived from mesenchymal stem cells (MSCs) have been reported to alleviate dysregulated inflammation and exert regenerative effects in osteoporosis therapy.^[^ ^21^ ^]^ Furthermore, small extracellular vesicles secreted by adipose‑derived stem cells (ADSCs) have been shown to promote bone regeneration by regulating macrophage polarization.^[^ ^22^ ^]^ Compared to MSCs isolated from bone marrow or umbilical cord, ADSCs can be easily obtained from a patient's abdominal fat aspirate, providing a more convenient and less invasive source to obtain small extracellular vesicles.^[^ ^23^ ^]^ Therefore, small extracellular vesicles derived from ADSCs (sEVs) are suitable chemical cues for endowing hydrogels with immunomodulation functions.

**Scheme 1:**
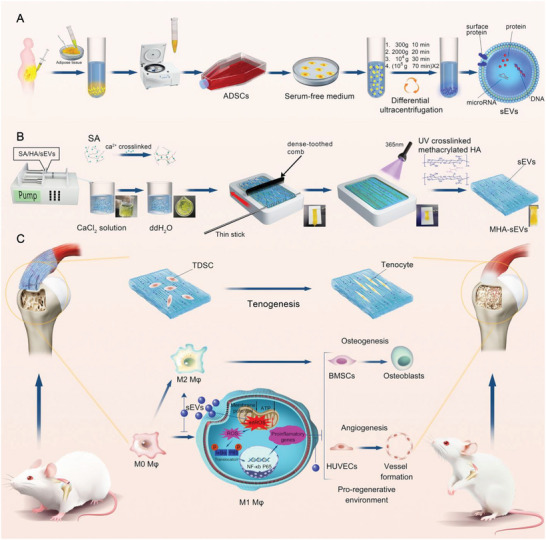
A) Isolation of sEVs. B) Fabrication of MHA‐sEVs. Macroscopic photos are shown in the lower right corner. C) Implantation of MHA‐sEVs at the TBI creates a regenerative microenvironment to promote TBH in a RCR model in osteoporotic rats.

We have fabricated a SA/HA macroporous hydrogel in our recent study.^[^ ^15^ ^]^ We hypothesize that this hydrogel has potential to induce tendon healing as SA has excellent biocompatibility, and HA is an essential component of tendon ECM shown to accelerate TBH following RCR.^[^ ^24^ ^]^ The macroporous structure of this SA/HA hydrogel can also benefit cell/tissue infiltration, while the addition of sEVs can help modulate Mφ polarization and alleviate rotator cuff tendinopathy.^[^ ^25^ ^]^ Based on our previous findings, we introduced an aligned hydrogel structure as well as sEVs in this study, with the aim of developing a SA/HA‐based macroporous hydrogel that has dual functions of an aligned architecture and immunomodulation ability for enhancing TBH, corresponding to the research strategy shown in **Scheme** **1**. We first isolated and characterized sEVs from human ADSCs (Scheme 1A). Then, SA/HA‐sEVs microfiber gels were produced using wet spinning, which were aligned using a comb and crosslinked to form a macroporous granular hydrogel with aligned microfiber gels and sEVs (MHA‐sEVs) (Scheme 1B). Meanwhile, macroporous granular hydrogels with randomly oriented microfiber gels and sEVs (MHR‐sEVs), macroporous granular hydrogels with aligned microfiber gels only (MHA), macroporous granular hydrogels with randomly oriented microfiber gels only (MHR), and NH were prepared for comparison. The hydrogels were characterized for morphology, structure, degradation, sEVs release behavior, and cytocompatibility before being implanted subcutaneously to investigate the effects of macroporous structure on cell/tissue infiltration and hydrogel‐tissue integration. The hydrogels were then implanted in a rat osteoporotic RCR model to investigate their therapeutic efficacy in TBH. To elucidate the mechanisms by which the hydrogels promoted TBH, their effects on tenogenic differentiation of human tendon‐derived stem cells (TDSCs) were investigated. Additionally, the effects of sEVs on M1 Mφ mitochondrial dysfunction and Mφ polarization, as well as the associated mechanisms were investigated (Scheme 1C).

## 2 Results

### 2.1 Characterization of ADSCs and sEVs

The identification of ADSCs is shown in Figure S1A–C (Supporting Information). Briefly, ADSCs used in this study showed a classic elongated spindle shape and were confirmed to have trilineage differentiation capacity, from the deposition of calcium nodules, proteoglycans, and lipid droplets after induction with osteogenic, chondrogenic, and adipogenic medium for 21 days, respectively. Flow cytometry showed that over 90% of ADSCs were positive for surface CD29, CD44, CD90, and CD105, while CD34 was predominantly negative.

sEVs derived from ADSCs were observed to be typical hollow vesicles with bilayer membranes by transmission electron microscopy (TEM). The particle size of sEVs ranged between 50 and 150 nm with an average size of 88.74 ± 22.29 nm. Western blot confirmed that the sEVs were enriched for CD9, TSG101, and HSP70 proteins, while calnexin was unexpressed (Figure S1D–F, Supporting Information). The sEVs could be internalized by recipient cells as significant amounts of sEVs were seen in Mφ, bone marrow‐derived mesenchymal stem cells (BMSCs), and human umbilical vein endothelial cells (HUVECs) after co‐incubation with the cells for 48 h (Figure S2, Supporting Information).

### 2.2 Preparation and Characterization of MHA‐sEVs

**Figure 1:**
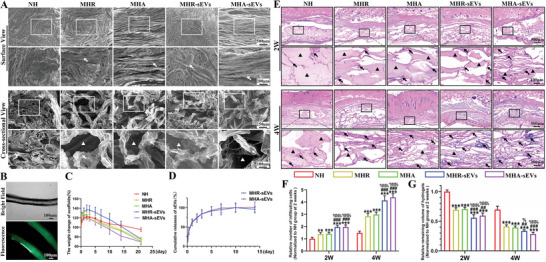
Fabrication and characterization of MHA‐sEVs. A) Surface view and cross‐sectional view of different hydrogel groups. The representative microfibers are marked with arrowheads, while micropores are marked with triangles. B) Morphology of microfibers. C) Degradation behavior of different hydrogels. D) Release characteristics of sEVs in MHR‐sEVs and MHA‐sEVs. E) Host tissue invasion of different hydrogels after rat subcutaneous implantation of hydrogels. The representative areas of cellular infiltration are marked with arrowheads. The remaining volume of hydrogels are marked with triangles. F–G) Semi‐quantitative analysis of the number of infiltrating cells in different hydrogels and the remaining volume of different hydrogels. ^**^ *p* < 0.01, ^***^ *p* < 0.001, when the data were compared with control group. ^##^ *p* < 0.01, ^###^ *p* < 0.001, when the data were compared with MHR group. ^%^ *p* < 0.05, ^%%%^ *p* < 0.001, when the data were compared with MHA group.

The method for preparing MHA‐sEVs hydrogels is shown in Scheme 1B and described in Experimental Section S1.5.1 (Supporting Information). In brief, SA/HA‐sEVs microfiber gels were prepared by wet spinning the SA/HA‐sEVs hydrogel precursor into CaCl_2_ solution, oriented with a close‐toothed comb, and finally cross‐linked by UV light to form MHA‐sEVs. The prepared hydrogels were freeze‐dried and observed with scanning electron microscopy (SEM) (**Figure** **1**A), showing clearly aligned microfiber structures (indicated by arrowheads) in MHA‐sEVs and MHA on the surface. In contrast, the microfibers in MHR‐sEVs and MHR were intertwined and randomly oriented. Microscale gaps and voids (indicated by triangles)were observed in all macroporous hydrogels, including MHA‐sEVs, MHR‐sEVs, MHA, and MHR, while no microfibers were observed in the NH. When the freeze‐dried hydrogels were observed from the cross‐sectional side, the MHA‐sEVs, MHR‐sEVs, MHA, and MHR all showed micron‐sized porous structures. In all hydrogels, the amount of uncrosslinked HA as well as their possible diffusion out of the hydrogel was expected to be minimal due to the rapid progression of calcium ion crosslinking of the SA hydrogel base structure, followed by further UV crosslinking of the SA/HA‐sEVs microfibers.

The microfiber gels had diameters of 104.55 ± 17.56 µm (Figure 1B). The degradation of macroporous hydrogels with sEVs was measured over 21 days of immersion in phosphate‐buffered saline (PBS) (Figure 1C). Among the hydrogel groups, NH showed the least mass at 4.99% ± 3.99%, far less than the other macroporous hydrogels. The MHR, MHA, MHR‐sEVs, and MHA‐sEVs hydrogels showed 25.85% ± 4.91%, 29.61% ± 4.85%, 24.82% ± 4.08%, and 30.68% ± 6.03% mass loss, respectively. There was no significant difference in degradation behavior among the four types of macroporous hydrogels, indicating that the alignment of microfiber gels and incorporation of sEVs did not affect the degradation rate. Additionally, the alignment of microfiber gels had no effect on the release behavior of sEVs, as burst release of sEVs was observed from both MHA‐sEVs and MHR‐sEVs in the first 3 days of incubation, resulting in a cumulative release of 80%. After 14 days, the cumulative release of sEVs reached ≈100% (Figure 1D).

All hydrogels showed good cytocompatibility as culture medium containing the hydrogel extracts had similar effects on the viability and proliferation of Mφ compared to normal culture medium (Figure S3, Supporting Information). After incubation with BMSCs for 5 days and HUVECs for 3 days, MHA‐sEVs and MHR‐sEVs significantly promoted the proliferation of BMSCs and HUVECs, while both MHA and MHR had no effect on these cells, suggesting that sEVs were capable of stimulating the growth of BMSCs and HUVECs (Figures S4 and S5, Supporting Information).

After subcutaneous implantation into rats, the hydrogel groups were evaluated by hematoxylin and eosin (H&E) staining of tissue/hydrogel (Figure 1E) and semi‐quantitative analysis of the number of infiltrating cells (Figure 1F) and remaining volume of hydrogels (Figure 1G). At 2 weeks, the NH group showed large blocks of dense hydrogel (indicated by the rectangular box) in the implantation area, accompanied by the infiltration of only a few cells (indicated by arrowheads) into the hydrogel boundary. In comparison, many voids were observed in the middle of the MHA and MHR hydrogels, and more cells (indicated by arrowheads) were seen in these voids, indicating that the macroporous microfiber structure of these hydrogels facilitated cell infiltration. The remaining volumes of MHR and MHA (indicated by black triangles) were smaller than those of the NH group. However, the MHA group with aligned microfiber gels did not show improved cell infiltration or a higher hydrogel degradation ratio compared to the MHR group. The addition of sEVs greatly improved cell infiltration and increased hydrogel degradation ratio in the MHA‐sEVs and MHR‐sEVs compared to MHA and MHR. At 4 weeks post‐implantation, all groups showed greater cell infiltration into the hydrogels compared to at 2 weeks. The NH group still had a clumpy dense structure, and few cells were observed inside the hydrogel. Meanwhile, the MHA and MHR groups showed significantly more infiltrated cells and lower remaining hydrogel volume than NH, while the MHA‐sEVs and MHR‐sEVs groups had the greatest amount of cell infiltration and smallest remaining hydrogel volume, indicating that the incorporation of sEVs can improve cell recruitment into the hydrogel and accelerate the degradation of hydrogels. Nevertheless, the alignment of microfibers did not affect cell infiltration as no significant differences were observed in the number of infiltrated cells between the MHA and MHR groups or the MHA‐sEVs and MHR‐sEVs groups (Figure 1F).

### 2.3 MHA‐sEVs Promotes Supraspinatus Tendon Repair in Osteoporotic RCR

Osteoporosis was successfully induced in female rats (Figure S6, Supporting Information). At 13 weeks after ovariectomy, significantly decreased bone volume (BV)/total volume (TV), trabecular number (Tb. N), and trabecular thickness (Tb.th) were observed in the distal femur and proximal humerus. The osteoporotic rats were used to construct an RCR model (Figure S7, Supporting Information). After RCR, different hydrogels were implanted in the experimental groups, while the control group received only direct suture but no hydrogel, and the sham group did not undergo RCR. Gross observation of the supraspinatus‐humerus complex at 2, 4, and 8 weeks of tendon repair showed no significant infection in any of the groups (Figure S8, Supporting Information). All groups showed no significant atrophy of the supraspinatus muscle during the postoperative period, but occasional fatty tissue was found surrounding the muscle in the RCR. Tissue hyperplasia was evident in all groups at 2 weeks after RCR, which subsided at 4 weeks and the repaired tendon contours could be identified in the MHA and MHA‐sEVs groups. At 8 weeks postoperation, the supraspinatus tendon morphology in the control and MHR groups was still not clearly defined. In contrast, the MHA, MHR‐sEVs, and MHA‐sEVs groups showed further improvements in tissue hyperplasia and more clearly defined tendon contours.

**Figure 2:**
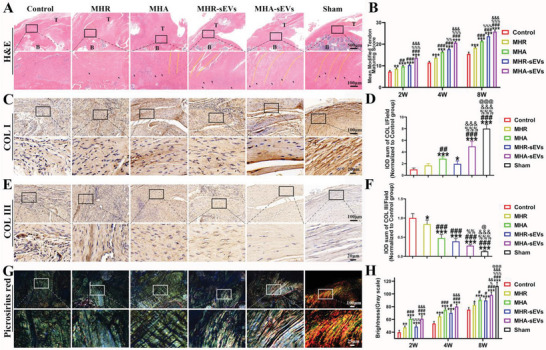
MHA‐sEVs facilitates supraspinatus tendon repair in a rat osteoporotic RCR model at 8 weeks. A) Representative H&E staining of the TBI. The representative fibrochondrocytes are marked with arrowheads. The aligned tendon fibers are indicated with yellow lines. B) Results of modified tendon maturity score of supraspinatus tendon postoperation. C,D) Representative immunohistochemical staining images and semi‐quantitative analysis of COL I in the tendon zone. E,F) Representative immunohistochemical staining images and semi‐quantitative analysis of COL III in the tendon zone. G,H) Representative picrosirius red staining of the TBI and semi‐quantification. B, bone; T, tendon. ^*^ *p* < 0.05, ^**^ *p* < 0.01, ^***^ *p* < 0.001, when the data were compared with control group. ^#^ *p* < 0.05, ^##^ *p* < 0.01, ^###^ *p* < 0.001, when the data were compared with MHR group. ^%^ *p* < 0.05, ^%%^ *p* < 0.01, ^%%%^ *p* < 0.001, when the data were compared with MHA group. ^&&^ *p* < 0.01, ^&&&^ *p* < 0.001, when the data were compared with MHR‐sEVs group. ^@^ *p* < 0.05, ^@@@^ *p* < 0.001, when the data were compared with MHA‐sEVs group.

The supraspinatus‐humerus complex tissue was extracted from the rat osteoporotic RCR model at different time points and stained with H&E (Figure S9A, Supporting Information (2 and 4 weeks) and **Figure** **2**A (8 weeks)). At 2 weeks after RCR, the regenerated tendon in all groups contained mostly fibrous tissue with increased cell density and neovascular tissue, likely due to the inflammatory response in the early stages of tendon healing. At 4 weeks, all groups showed reduced cell density in the regenerated tendon, with the highest cell density observed in the control group. At 8 weeks, cell density continued to decrease in all groups (Figure 2A). The tendon‐to‐bone interface (TBI) (indicated by rectangular box) was inferior in the control group compared to the hydrogel groups, with poor tendon‐to‐bone integration separated by an apparent boundary. Hydrogels with aligned structure showed improved tendon‐to‐bone integration, where more aligned tendon fibers were attached to the bone (indicated with yellow lines) in the MHA and MHA‐sEVs groups compared to the MHR and MHR‐sEVs groups. Furthermore, sEVs improved tendon‐to‐bone attachment as more fibrocartilage‐like tissue (indicated with black arrowheads) was observed at the TBI in the MHR‐sEVs and MHA‐sEVs groups than in the MHR and MHA, with the MHA‐sEVs group showing the most effective tendon‐to‐bone attachment.

At 8 weeks post‐implantation, supraspinatus tendon repair in different groups was also assessed by immunohistochemical staining for COL I and COL III (Figure 2C–F). More abundant COL I than COL III indicates good tendon repair.^[^ ^26^ ^]^ The tendon zone of the TBI (indicated by rectangular box) showed higher COL I content and lower COL III in the hydrogel groups compared to the control. The presence of aligned cues in MHA and MHA‐sEVs compared to MHR and MHR‐sEVs, as well as sEVs in MHA‐sEVs compared to MHA also separately led to increased COL I and decreased COL III. MHA‐sEVs demonstrated the most effective tendon repair as it showed the highest COL I/COL III ratio among all groups.

Representative images of picrosirius red staining show COL I as yellow or red and COL III as green under polarized light microscopy.^[^ ^27^ ^]^ At 2 weeks, a disorganized collagen arrangement was observed in all groups, while at 4 and 8 weeks, the tendon fibers became more organized (Figure 2G; Figure S9B, Supporting Information). The aligned microstructure of MHA and MHA‐sEVs better‐facilitated tendon reconstruction compared to MHR and MHR‐sEVs, shown by more aligned collagen arrangement and COL I content. The incorporation of sEVs also improved collagen organization and COL I content in MHR‐sEVs and MHA‐sEVs compared to MHR and MHA. The semi‐quantitative scoring of picrosirius red staining was consistent with these observations (Figure 2H). The grayscale value gradually increased in all groups from 2 to 8 weeks after surgery, although the control group improved more slowly compared to the other groups. At all postoperative time points, the grayscale value of the MHA and MHA‐sEVs groups was consistently better than MHR and MHR‐sEVs, suggesting that the oriented hydrogel structure improved the quality of tendon regeneration. The effect of sEVs on tendon repair was not obvious between MHR and MHR‐sEVs at 2 weeks, or between MHA and MHA‐sEVs at 2 and 4 weeks. However, superior tendon repair was observed in MHR‐sEVs and MHA‐sEVs at 8 weeks compared to MHR and MHA. The MHA‐sEVs group scored the highest among all groups, although it still fell short of the sham group.

The histological state of the regenerated supraspinatus tendon improved with time in all groups. The details of the semi‐quantitative tendon maturation scoring system are shown in Table S4 (Supporting Information). At all postoperative time points, the hydrogel groups had higher tendon maturation scores compared to the control group (Figure 2B). Other trends were similar to those described above for histological and immunohistochemical results, whereby an aligned hydrogel structure as well as incorporation of sEVs led to higher tendon maturation scores, and the MHA‐sEVs group achieved the highest scores among all groups.

### 2.4 MHA‐sEVs Regulates Inflammatory Microenvironment for Bone Regeneration in Osteoporotic RCR

**Figure 3:**
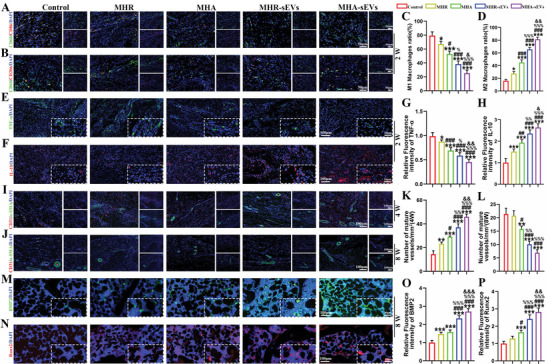
MHA‐sEVs regulates Mφ polarization and secretion of inflammation cytokines to facilitate mature vessel formation and bone regeneration at the TBI. Representative images and semi‐quantitative analysis are shown for immunofluorescence staining of A,C) CD68 (green) and CD86 (red); B,D) CD68 (green) and CD206 (red); E,G) TNF‐α (green); F,H) IL‐10 (red); I,L) α‐SMA (green) and CD31 (red); M,O) BMP2 (green); N,P) Runx2 (red). ^*^ *p* < 0.05, ^**^ *p* < 0.01, ^***^ *p* < 0.001, when the data were compared with control group. ^#^ *p* < 0.05, ^##^ *p* < 0.01, ^###^ *p* < 0.001, when the data were compared with MHR group. ^%^ *p* < 0.05, ^%%^ *p* < 0.01, ^%%%^ *p* < 0.001, when the data were compared with MHA group. ^&^ *p* < 0.05, ^&&^ *p* < 0.01, ^&&&^ *p* < 0.001, when the data were compared with MHR‐sEVs group.

TBI tissue in different groups at 2 weeks postoperation were co‐immunostained with CD68/CD86 (**Figure** **3**A,C) and CD68/CD206 (Figure 3B,D), as well as immunostained with tumor necrosis factor‐alpha (TNF‐α) (Figure 3E,G) and anti‐inflammatory factor interleukin‐10 (IL‐10) (Figure 3F,H) to identify M1 and M2 Mφ. M1 polarized Mφ are indicated by high expression of CD86 and TNF‐α, while M2 polarized Mφ are indicated by high expression of CD206 and IL‐10. Hydrogel groups with better ability to promote tissue regeneration and immunomodulation are expected to stimulate Mφ polarization to M2 Mφ that have a pro‐regeneration phenotype. The control group showed the highest number of M1 Mφ and the lowest number of M2 Mφ, indicating an acute inflammatory response in the surgical area. The hydrogel groups showed varying degrees of immunomodulatory ability, but all reduced the level of acute inflammation compared to the control group. Hydrogels with aligned microfiber gels (MHA and MHA‐sEVs) and incorporating sEVs (MHR‐sEVs and MHA‐sEVs) showed stronger immunomodulatory ability compared to their counterparts. Meanwhile, the MHA‐sEVs group showed the best ability to modulate Mφ polarization toward the pro‐regenerative M2 phenotype.

Vascular regeneration at the TBI was detected by co‐immunostaining with CD31/α‐smooth muscle actin (α‐SMA) at 4 (Figure 3I,K) and 8 weeks (Figure 3J,L) after RCR. At 4 weeks, increased blood vessel formation was observed in all hydrogel groups compared to the control group. Neovascularization was enhanced in the hydrogel groups containing aligned microfiber gels (MHA and MHA‐sEVs) and sEVs (MHR‐sEVs and MHA‐sEVs), with MHA‐sEVs displaying the highest level of neovascularization among all groups. At 8 weeks, the total number of mature vessels in the hydrogel‐implanted groups was lower than at 4 weeks. Similar numbers of vessels were found in the control and MHR groups, which were higher than those observed in the MHA, MHR‐sEVs, and MHA‐sEVs groups. No significant differences were noted between MHR‐sEVs and MHA‐sEVs at 8 weeks, both of which showed the greatest reduction in the expression of mature blood vessels compared to other groups. The MHA‐sEVs group showed the greatest pro‐angiogenic effect at 4 weeks, followed by the most prominent inhibitory effect on vessel formation at 8 weeks.

The status of bone regeneration in different groups was assessed at 8 weeks after RCR by immunofluorescence assays of bone morphogenetic protein (BMP2; Figure 3M) and runt‐related transcription factor 2 (Runx2; Figure 3N), together with semi‐quantitative analysis (Figure 3O,P). Compared to the control group, BMP2 expression was significantly higher in all hydrogel groups while Runx2 expression was significantly higher in the MHA, MHR‐sEVs, and MHA‐sEVs groups. Hydrogels with aligned structure benefited bone formation, as seen through higher Runx2 expression in MHA and MHA‐sEVs compared to MHR and MHR‐sEVs, as well as higher BMP2 expression in MHA‐sEVs compared to MHR‐sEVs. Moreover, the MHR‐sEVs and MHA‐sEVs groups containing sEVs enhanced bone regeneration compared to MHR and MHA, as seen through higher expression of both BMP2 and Runx2. The highest expression of both osteogenic factors was seen in the MHA‐sEVs group. The status of bone repair status at 8 weeks was also assessed by immunohistochemical staining of COL I and COL III, together with semi‐quantitative analysis (Figure S10, Supporting Information). Compared to MHR and MHA, the incorporation of sEVs in MHR‐sEVs and MHA‐sEVs contributed to higher COL I and lower COL III expression.

**Figure 4:**
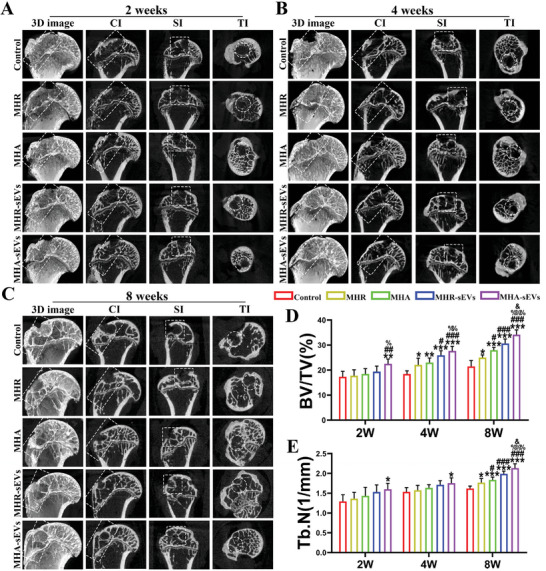
MHA‐sEVs enhance new bone formation in the bone tunnel of the humeral head. A–C) Representative 3D reconstruction, coronal, sagittal, and transverse μ‐CT images at 2, 4, and 8 weeks of tendon repair. D,E) Semi‐quantitative analysis of BV/TV and Tb.N of regenerated bone tissue. The bone tunnel area was circled by white dotted lines. CI, coronal images; SI, sagittal images; TI, transverse images. ^*^ *p* < 0.05, ^**^ *p* < 0.01, ^***^ *p* < 0.001, when the data were compared with control group. ^#^ *p* < 0.05, ^##^ *p* < 0.01, ^###^ *p* < 0.001, when the data were compared with MHR group. ^%^ *p* < 0.05, ^%%^ *p* < 0.01, ^%%%^ *p* < 0.001, when the data were compared with MHA group. ^&^ *p* < 0.05, when the data were compared with MHR‐sEVs group.

New bone formation in the bone tunnel of the regenerated humeral head at 2, 4, and 8 weeks postoperation was further assessed by micro‐computed tomography (µ‐CT) (**Figure** **4**A–C) with semi‐quantitative analysis of bone volume/total volume fraction (BV/TV) and trabecular number (Tb. N) (Figure 4D,E), as well as trabecular thickness (Tb. th) and trabecular separation (Tb. sp) (Figure S11, Supporting Information). The bone tunnel was evident in all groups at 2 weeks postoperation. The MHA‐sEVs group showed significantly higher BV/TV (22.52% ± 1.9%) compared to the control (17.32 ± 2.19%), MHR(17.76% ± 2.35%), and MHA (18.48% ± 2.1%) groups, as well as significantly higher Tb.N (1.6 ± 0.15 mm^−1^) than the control (1.29 ± 0.17 mm^−1^). At 4 weeks, the BV/TV in all hydrogel groups was considerably higher than the control (18.47% ± 1.25% for Control, 22.09% ± 2.66% for MHR, 23% ± 1.83% for MHA, 25.93% ± 1.8% for MHR‐sEVs, 27.68% ± 1.84% for MHA‐sEVs,). Meanwhile, the MHA‐sEVs group showed higher Tb. N (1.75 ± 0.13 mm^−1^) compared to the control (1.53 ± 0.11 mm^−1^), and lower Tb.sp (0.26 ± 0.01 mm) compared to the control (0.31 ± 0.02 mm), MHR (0.3 ± 0.01 mm), and MHA groups (0.29 ± 0.02 mm). At 8 weeks, further narrowing of the bone tunnel was observed in all groups. The BV/TV and Tb. N of hydrogel implanted groups were higher than the control. MHA (27.83% ± 1.33%) and MHA‐sEVs (34.15% ± 1.92%) with aligned hydrogel structure showed higher BV/TV than MHR (25.03% ± 1.02%) and MHR‐sEVs (30.65% ± 1.79%), while MHA‐sEVs (2.14 ± 0.11 1/mm) also showed higher Tb.N than MHR‐sEVs (1.99 ± 0.07 mm^−1^). MHR‐sEVs and MHA‐sEVs incorporating sEVs showed higher BV/TV, Tb. N, and Tb.th, and lower Tb.sp than MHR and MHA. Taken together, the MHA‐sEVs group demonstrated the best overall bone regeneration.

### 2.5 MHA‐sEVs Promotes TBI Reconstruction with Enhanced Biomechanical Strength

**Figure 5:**
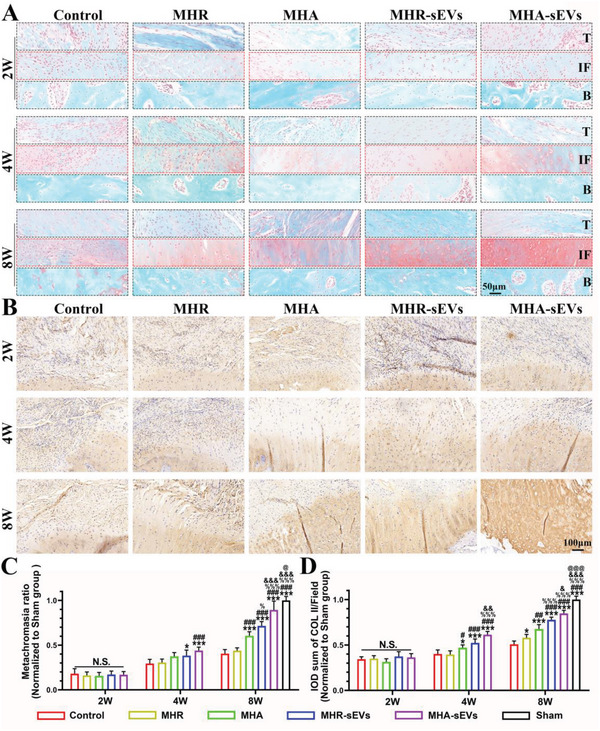
MHA‐sEVs promote fibrocartilage regeneration in the TBI. A) Representative images of Safranin O‐fast green staining. B) COL II immunohistochemical staining. C) Semi‐quantitative analysis of Safranin O‐fast green staining. D) Semi‐quantitative analysis of COL II immunohistochemical staining. T, tendon; IF, interface; B, bone. ^*^ *p* < 0.05, ^***^ *p* < 0.001, when the data were compared with control group. ^#^ *p* < 0.05, ^##^ *p* < 0.01, ^###^ *p* < 0.001, when the data were compared with MHR group. ^%^ *p* < 0.05, ^%%%^ *p* < 0.001, when the data were compared with MHA group. ^&^ *p* < 0.05, ^&&^ *p* < 0.01,^&&&^ *p* < 0.001, when the data were compared with MHR‐sEVs group. ^@^ *p* < 0.05, ^@@@^ *p* < 0.001, when the data were compared with MHA‐sEVs group.

Regeneration of fibrocartilage at the TBI was evaluated with safranin O‐fast green staining and collagen type II (COL II) immunohistochemistry (**Figure** **5**A,B; Figure S12, Supporting Information), together with semi‐quantitative analysis of the positively stained areas (Figure 5C,D). The dry fraction of cartilage ECM consists of proteoglycan and COL II, whereby proteoglycan in fibrocartilage is stained red by safranin O. No obvious fibrocartilage formation was observed in any of the groups at 2 weeks postoperation. After 4 weeks, the safranin O‐positive and COL II‐positive areas were larger in the MHR‐sEVs and MHA‐sEVs groups compared to the control, as well as larger in MHA‐sEVs compared to MHR. Meanwhile, hydrogels with aligned microfiber gels (MHA and MHA‐sEVs) and containing sEVs (MHR‐sEVs and MHA‐sEVs) exhibited larger COL II‐positive areas than their counterparts. At 8 weeks, hydrogel groups with aligned structure and sEVs incorporation showed higher stimulatory effects on fibrocartilage regeneration as seen through increased safranin O and COL II staining. The integrated optical density (IOD) of the COL II in each field was normalized to the sham group and the relative value for Control, MHR, MHA, MHR‐sEVs, and MHA‐sEVs at 8 weeks were 0.52 ± 0.03, 0.59 ± 0.03, 0.69 ± 0.04, 0.78 ± 0.02, and 0.85 ± 0.04, respectively. Notably, the greatest fibrocartilage area was seen in the MHA‐sEVs group, which exceeded that of all other groups and reached 80–90% of the sham group.

**Figure 6:**
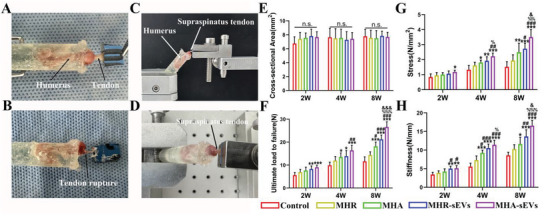
MHA‐sEVs increase the biomechanical strength of the supraspinatus‐humerus complex. A) Sample preparation before biomechanical tests. B) Tendon rupture scene after biomechanical testing. C,D) Biomechanical test scenarios. E) Cross‐sectional area. F) Ultimate load to failure. G) Stress. H) Stiffness. *p < 0.05, **p < 0.01, ***p < 0.001, when the data were compared with control group. ^#^ *p* < 0.05, ^##^ *p* < 0.01, ^###^ *p* < 0.001, when the data were compared with MHR group. ^%^ *p* < 0.05, ^%%^ *p* < 0.01, ^%%%^ *p* < 0.001, when the data were compared with MHA group. ^&^ *p* < 0.05, ^&&&^ *p* < 0.001, when the data were compared with MHR‐sEVs group.

Biomechanical testing was conducted on the different groups of regenerated supraspinatus‐humerus complex (**Figure** **6**A–D), and their biomechanical strength was computed (Figure 6E–H). At all postoperative time points, the groups showed no significant differences in the cross‐sectional surface area of the supraspinatus tendon, and the surface area also did not change with regeneration time. From 2 to 8 weeks postoperation, the biomechanical strength of the complex increased in all groups, including the ultimate load to failure, stress, and stiffness. At 2 weeks, the MHR‐sEVs and MHA‐sEVs groups showed higher ultimate load to failure and stiffness than the control, while MHA‐sEVs also showed higher stress than the control (ultimate load to failure (N): 5.58 ± 1.24 for Control, 8.25 ± 1.38 for MHR‐sEVs, 9.02 ± 0.98 for MHA‐sEVs; stiffness (N mm^−1^): 3.42 ± 0.6 for Control, 5 ± 0.67 for MHR‐sEVs, 5.12 ± 0.68 for MHA‐sEVs; stress (N mm^−2^): 0.84 ± 0.22 for Control, 1.18 ± 0.12 for MHA‐sEVs). At 4 weeks, the MHA, MHR‐sEVs, and MHA‐sEVs groups all showed greater ultimate load to failure, stress, and stiffness than the control. Among hydrogel groups, MHA had higher stiffness than MHR, MHA‐sEVs had higher stress than MHA, and MHR‐sEVs and MHA‐sEVs had higher stiffness than MHR and MHA (ultimate load to failure (N): 9.91 ± 1.37 for Control, 11.94 ± 1.83 for MHR, 13.48 ± 1.83 for MHA, 13.86 ± 2.47 for MHR‐sEVs, 16.4 ± 2.25 for MHA‐sEVs; stress (N mm^−2^): 1.33 ± 0.28 for Control, 1.62 ± 0.21 for MHR, 1.8 ± 0.16 for MHA, 1.92 ± 0.27 for MHR‐sEVs, 2.22 ± 0.21 for MHA‐sEVs; stiffness (N mm^−1^): 5.54 ± 0.95 for Control, 7.3 ± 1.12 for MHR, 9.22 ± 0.76 for MHA, 10.52 ± 1.09 for MHR‐sEVs, 11.39 ± 1.26 for MHA‐sEVs). At 8 weeks, the same trends were observed between the control and hydrogel groups as at 4 weeks. MHA and MHA‐sEVs with aligned microstructure had a higher ultimate load to failure than MHR and MHR‐sEVs, while MHA‐sEVs showed higher stiffness and stress than MHR‐sEVs. In addition, the MHR‐sEVs and MHA‐sEVs hydrogel groups containing sEVs showed greater ultimate load to failure, stress, and stiffness than MHR and MHA (ultimate load to failure (N): 11.61 ± 1.96 for Control, 14.35 ± 1.44 for MHR, 18.11 ± 1.93 for MHA, 21.2 ± 1.9 for MHR‐sEVs, 26.59 ± 2.28 for MHA‐sEVs; stress (N mm^−2^): 1.52 ± 0.34 for Control, 1.94 ± 0.38 for MHR, 2.49 ± 0.61 for MHA, 2.72 ± 0.36 for MHR‐sEVs, 3.5 ± 0.5 for MHA‐sEVs; stiffness (N mm^−1^): 8.57 ± 0.85 for Control, 10.26 ± 1.18 for MHR, 11.58 ± 1.86 for MHA, 13.64 ± 1.8 for MHR‐sEVs, 16.55 ± 1.45 for MHA‐sEVs). The highest overall biomechanical strength was observed in the MHA‐sEVs group, reaching about twice that of the control group.

At 8 weeks after surgery, the results of routine blood tests and liver and kidney function tests for all experimental groups were not significantly different from those of sham rats (Figure S13, Supporting Information). Pathological analysis of the major organs of rats in all experimental groups, including the heart, liver, spleen, lung, and kidney, revealed no obvious adverse effects associated with hydrogel implantation (Figure S14, Supporting Information). MHA‐sEVs are therefore considered biologically safe following in vivo implantation.

### 2.6 MHA‐sEVs Enhanced Tenogenic Differentiation of TDSCs In Vitro

**Figure 7:**
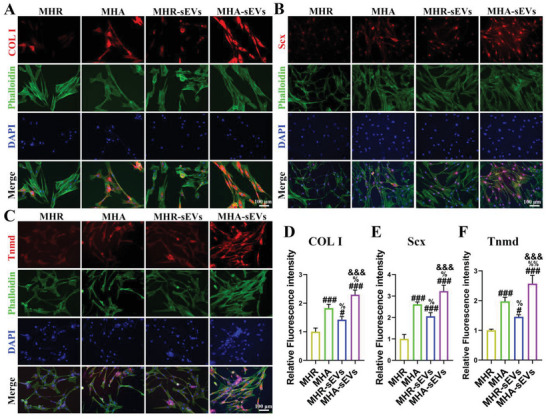
MHA‐sEVs enhance the tenogenesis of TDSCs. A–C) Immunofluorescence detection of the expression of COL I, Scx, and Tnmd after TDSCs were seeded on various hydrogels. D–F) Semi‐quantitative analysis of immunofluorescence staining. ^#^ *p* < 0.05, ^###^ *p* < 0.001 when the data were compared with the MHR group. ^%^ *p* < 0.05, ^%%^ *p* < 0.01 when the data were compared with the MHA group. ^&&&^ *p* < 0.001 when the data were compared with the MHR‐sEVs group.

When TDSCs were cultured on different hydrogels, the cells cultured on MHA showed much higher expression of tenogenesis‐associated proteins COL I, scleraxis (Scx), and Tnmd than those cultured on MHR, indicating that hydrogels with aligned microfiber gels stimulated tenogenic differentiation in TDSCs. The incorporation of sEVs in the hydrogels further enhanced these stimulatory effects as TDSCs cultured on MHA‐sEVs and MHR‐sEVs expressed more COL I, Scx, and Tnmd than those cultured on MHA and MHR. The MHA‐sEVs showed the strongest effects on inducing tenogenic differentiation of TDSCs among all hydrogel groups (**Figure** **7**).

### 2.7 Mechanisms Underlying the Immunomodulatory Effects of sEVs on Osteogenesis and Angiogenesis

#### 2.7.1 The Effects of sEVs on the Mitochondrial Dysfunction of M1 Mφ

**Figure 8:**
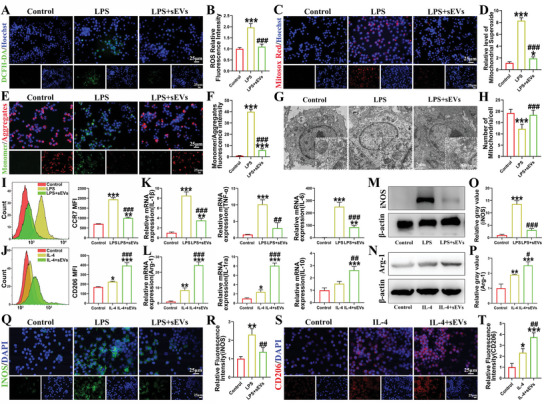
sEVs improve mitochondrial dysfunction of M1 Mφ and regulate Mφ polarization. A–D) Representative images and semi‐quantitative analysis of intracellular ROS and mitochondrial superoxide. E,F) Representative images and semi‐quantitative analysis of ΔΨm detected by JC‐1 probe. G,H) Representative images of Mφ mitochondria under TEM and semi‐quantitative analysis of mitochondria number. I,J) Flow cytometry and semi‐quantitative analysis of Mφ surface markers (CCR7 and CD206). K,L) mRNA levels analyzed by RT‐qPCR. M‐P) Protein levels (iNOS and Arg‐1) analyzed by western blot and semi‐quantification. Q–T) Protein levels (iNOS and CD206) analyzed by immunofluorescence and semi‐quantification. ^*^ *p* < 0.05, ^**^ *p* < 0.01, ^***^ *p* < 0.001, when the data were compared with control group. ^#^ *p* < 0.05, ^##^ *p* < 0.01, ^###^ *p* < 0.001, when the data were compared with LPS or IL‐4 group.

Mφ activated with LPS treatment showed a significant increase in their levels of intracellular ROS shown by DCFH‐DA staining (**Figure** **8**A,B) and mitochondrial superoxide shown by mitosox red staining (Figure 8C,D) compared to non‐activated Mφ, indicating that the LPS treatment had an acute inflammatory effect. When the activated Mφ were cultured with sEVs, these inflammatory effects were profoundly inhibited as reflected by a dramatic decrease in the levels of intracellular ROS and mitochondrial superoxide matching those of non‐activated Mφ. The mitochondrial membrane potential (ΔΨm), which is a driving force for ATP production, can be detected using the JC‐1 probe. A significant reduction in monomer/aggregate ratio was observed when sEVs were added to LPS‐treated Mφ, which was reflective of an increase in ΔΨm (Figure 8E,F). Direct measurement of ATP showed reduced concentration in M1 Mφ after LPS stimulation, which was restored by adding sEVs (Figure S15, Supporting Information). TEM imaging following LPS stimulation also revealed changes in mitochondrial morphology, where M1 Mφ showed a significant reduction in the number of mitochondria and mitochondrial cristae compared to M0 Mφ. The addition of sEVs resulted in the restoration of the mitochondrial parameters to levels similar to control non‐activated Mφ (Figure 8G,H).

#### 2.7.2 The Effects of sEVs on the Polarization of Mφ and Associated Mechanisms

The addition of sEVs to LPS‐treated Mφ inhibited M1 Mφ‐associated C‐C chemokine receptor type 7 (CCR7) expression and promoted M2 Mφ‐associated CD206 expression (Figure 8I,J). Similar results were observed when M1/M2 Mφ‐related gene expression was analyzed by real‐time quantitative polymerase chain reaction (RT‐qPCR). The addition of sEVs inhibited M1 Mφ‐associated IL‐1β, TNF‐α, and IL‐6 gene expression and increased M2 Mφ‐associated Arginase 1 (Arg‐1), IL‐1ra, and IL‐10 expression (Figure 8K,L). Protein detection also showed that the sEVs inhibited M1 Mφ‐associated inducible nitric oxide synthase (iNOS) expression (Figure 8m,O,Q,R) while promoting M2 Mφ‐associated Arg‐1 (Figure 8N,P) and CD206 expression (Figure 8S,T).

**Figure 9:**
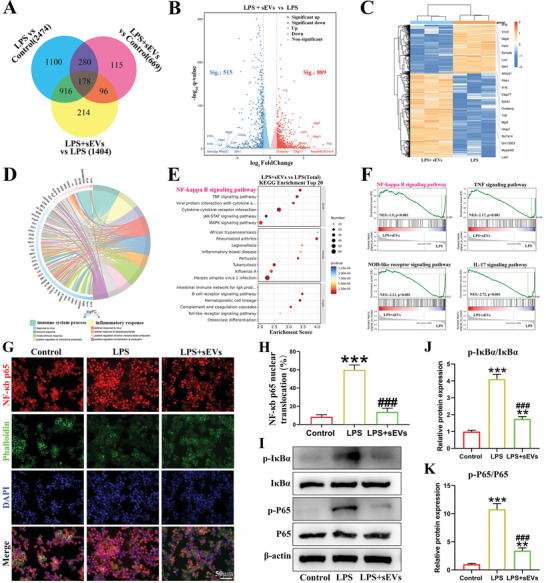
Transcriptome sequencing, immunofluorescence, and western blot to analyze the mechanisms by which sEVs regulate M1 Mφ. A) Venn plot showing differential gene expression among groups. B,C) Volcano map and heat map of differentially expressed genes between LPS + sEVs and LPS. D,E) GO and KEGG enrichment analysis of differentially expressed genes and pathways. F) GSEA analysis of NF‐κB, TNF, NOD‐like receptor, and IL‐17 signaling pathways. G,H) Immunofluorescence staining and semi‐quantitative analysis for NF‐κb p65 translocation into the nucleus. I–K) Western blot and semi‐quantitative analysis of NF‐κb pathway‐related protein expression (p‐IκBα, IκBα, p‐P65, and P65). ^**^ *p* < 0.01, ^***^ *p* < 0.001, when the data were compared with control group. ^###^ *p* < 0.001, when the data were compared with LPS.

RNA‐sequencing of Mφ before and after LPS treatment, and after sEVs treatment was performed to investigate major genes and signaling pathways regulated by the sEVs. No treatment was administered to the control group. Principal component analysis (PCA) showed significant differences in RNA expression among the control (non‐activated Mφ), LPS, and LPS + sEVs groups (Figure S16, Supporting Information). There was a total of 2474 differentially expressed genes between the LPS and control groups, and 1404 between the LPS and LPS + sEVs groups (**Figure** **9**A). Differential gene expression between the LPS and control groups is illustrated by a volcano map and heat map (Figure S17A,B, Supporting Information). GO enrichment analysis showed that these differentially expressed genes were mainly related to the cellular response to LPS, immune response, and inflammatory response, which were consistent with the treatment conditions (Figure S17C, Supporting Information). KEGG analysis showed that these genes were mainly associated with inflammatory pathways, such as the TNF and nuclear factor‐kappaB (NF‐κb) signaling pathways (Figure S17D, Supporting Information). Gene set enrichment analysis (GSEA) also verified that the TNF, NF‐κb, IL‐17, and NOD‐like receptor inflammation‐related signaling pathways were up‐regulated in the LPS group (Figure S17E–H, Supporting Information). These results confirm that M0 Mφ transformed into M1 Mφ following LPS stimulation, accompanied by the upregulation of inflammation‐associated genes and pathways. Significant differences in gene expression were observed between the LPS + sEVs and LPS groups as shown by the volcano and heat map plots (Figure 9B,C). GO analysis revealed that the differentially expressed genes were primarily associated with the immune system process and inflammatory response, while KEGG analysis indicated associations with the NF‐κb signaling pathway (Figure 9D,E). GSEA results verified that the addition of sEVs significantly downregulated the NF‐κb, TNF, NOD‐like receptor, and IL‐17 signaling pathways in Mφ (Figure 9F).

Nuclear localization is a prerequisite for NF‐κb p65 phosphorylation, which activates NF‐κb signaling. Immunofluorescence staining showed that the number of NF‐κb p65 nuclear‐localized Mφ after LPS stimulation was about seven times higher than that in control M0 Mφ, but the addition of sEVs returned the number of these cells similar to the control (Figure 9G,H). Meanwhile, western blot showed that p‐IκBα and p‐P65 protein expression in M1 Mφ were significantly reduced after the addition of sEVs (Figure 9I–K). These findings suggest that sEVs can improve the inflammatory microenvironment mediated by M1 Mφ through NF‐κb signaling.

#### 2.7.3 The Effects of sEVs on Osteogenic Differentiation of BMSCs and Angiogenesis of HUVECs by Modulating Mφ Polarization

**Figure 10:**
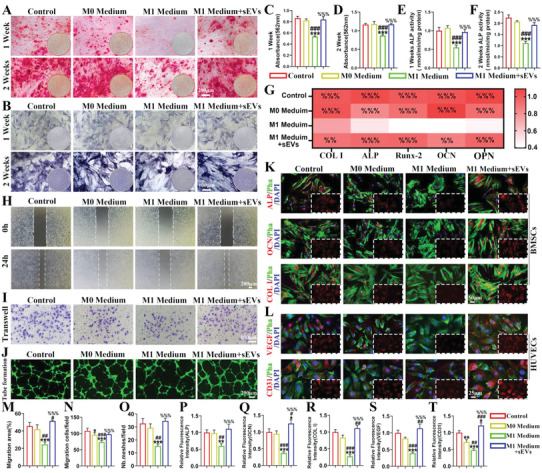
sEVs alleviate M1 Mφ‐mediated inflammatory microenvironment to promote osteogenesis and angiogenesis. After 1 and 2 weeks of osteogenic induction in BMSCs, A,C,D) Alizarin red staining and semi‐quantitative analysis; B,E,F) ALP staining and semi‐quantitative analysis; G) Expression levels of osteogenesis‐related genes (COL‐1, ALP, RUNX‐2, OCN, and OPN) measured by RT‐qPCR; K) Expression levels of osteogenesis‐related proteins (ALP, OCN, and COL I) detected by immunofluorescence assay. For analysis of angiogenesis by HUVECs, H,I,M,N) representative images and semi‐quantitative analysis of migration and transwell experiments; J,O) Representative images and semi‐quantitative analysis of tube formation; L) Expression levels of angiogensis‐related proteins (VEGF and CD31) detected by immunofluorescence assay. P–T) Semi‐quantitative analysis of immunofluorescence assays. ^*^ *p* < 0.05, ^**^ *p* < 0.01, ^***^ *p* < 0.001, when the data were compared with control group. ^#^ *p* < 0.05, ^##^ *p* < 0.01, ^###^ *p* < 0.001, when the data were compared with M0 Meduim group. ^%%^ *p* < 0.001, ^%%%^ *p* < 0.001, when the data were compared with M1 Meduim group.

The role of sEVs in modulating osteogenic differentiation and angiogenesis was investigated through in vitro culture of BMSCs and HUVECs using Mφ conditioned medium. BMSCs were used for osteogenesis, which was treated with an osteogenic medium mixed with different types of Mφ conditioned medium (M0, M1, and M1 + sEVs). In the M1 Medium group, the markers of osteogenic differentiation in BMSCs were significantly reduced after 1 and 2 weeks of culture compared to the control and M0 Medium groups, including calcium nodule formation (**Figure** **10**A,C,D) and ALP activity (Figure 10B,E,F), but this inhibitory effect was largely attenuated in the M1 + sEVs group. Similarly, RT‐qPCR analysis showed the suppression of osteogenic gene expression (COL I, ALP, Runx‐2, osteocalcin (OCN), and osteopontin (OPN)) in the M1 group, which was largely reversed in the M1 + sEVs group (Figure 10G). Immunofluorescence assays verified the inhibition of osteogenesis‐related protein expression (ALP, OCN, COL I) in the M1 group, while the M1 + sEVs group restored these protein levels to match (ALP, COL I) or even supersede (OCN) the control (Figure 10K,P–R).

HUVECs were used for angiogenesis experiments, which were grown in endothelial cell medium mixed with different types of Mφ conditioned medium (M0, M1, and M1 + sEVs). HUVEC migration (Figure 10H,I,M,N) and tube formation (Figure 10J,O) were both significantly inhibited in the M1 Medium group, while the M1 + sEVs Medium group showed restoration of these angiogenic activities to the same level as the control. The expression levels of angiogenesis‐related proteins (VEGF, CD31) in HUVECs followed a similar trend as shown by immunofluorescence assay, where the reduced expression of these proteins seen in M1 Medium was completely restored in M1 + sEVs (VEGF) or even to a higher level than the control (CD31) (Figure 10L,S,T). These results collectively suggest that the sEVs have trophic functions, which can help facilitate both osteogenesis and angiogenesis during TBH by modulating the inflammatory microenvironment.

## 3 Discussion

Macroporous hydrogels are gaining increasing attention in tissue regeneration due to their advantageous structural properties. Limited studies on macroporous hydrogels with aligned architecture have pointed to their beneficial properties for promoting osteoporotic TBH.^[^ ^28^ ^]^ To overcome the drawbacks of current preparation techniques, this study developed an effective, simple, and practical method to synthesize a macroporous hydrogel (MHA‐sEVs) with aligned architecture and immunomodulatory properties by assembling microfiber gels and incorporating sEVs. The MHA‐sEVs could enhance cell infiltration into the hydrogel through its macroporous structure, facilitate supraspinatus tendon repair through its aligned architecture, and accelerate bone regeneration by modulating the inflammatory microenvironment. By simultaneously promoting tendon and bone regeneration, MHA‐sEVs enhanced the reconstruction of fibrocartilage at the TBI and ultimately improved the biomechanical strength of the supraspinatus‐humerus complex, which is essential for reducing RCR retear rates.

Existing methods for preparing macroporous hydrogels with an aligned structure are limited, and experience drawbacks including cumbersome synthesis steps, requirement for sophisticated equipment, high costs, possible toxic chemical residues, and harsh experimental conditions such as high/low temperatures or cytotoxic solvents.^[^ ^12^, ^17^, ^29^ ^]^ This study provides a broadly applicable, effective strategy for fabricating high‐aspect‐ratio microfibre gels, and a straightforward and simple method for assembling these into macroporous hydrogels with aligned architecture. Ionic cross‐linkable materials such as SA, and UV cross‐linkable biomaterials such as methylated HA and gelatin methacrylate are typical biomaterials used for fabricating microgels.^[^ ^30^ ^]^ However, these materials are usually used alone to produce spherical or low‐aspect‐ratio microgels that exist in the form of jammed microgels within the bulk macroporous hydrogel. As there are no chemical interactions between the jammed microgels, the resulting hydrogel typically has poor shape fidelity and mechanical stability.^[^ ^14^, ^31^ ^]^ Moreover, spherical microgels can only provide homogeneous cues to cells in all directions and cannot supply important guidance cues to cells of anisotropic tissues, such as muscle and tendon whose properties vary in three dimensions.^[^ ^12^, ^14^ ^]^ In this study, we creatively used a binary component system to fabricate the microgels with wet‐spinning technology. This strategy is applicable to any binary material system where the two components are crosslinked using different methods. In addition, wet spinning can easily produce monodisperse high‐aspect‐ratio microgels with different geometries in a tightly controlled and continuous manner, which is highly desirable for fabricating macroporous hydrogels with aligned architecture for anisotropic tissues such as tendons. The MHA‐sEVs in this study was fabricated from commercial reagents and biomaterials, including SA, HA, and CaCl_2_, as well as sEVs that can be easily isolated from abdominal adipose tissue with minimal trauma. These synthesis materials all exhibit excellent biocompatibility and are easily accessible. The fabrication method for MHA‐sEVs also does not require sophisticated equipment, involving mainly a syringe pump, plastic molds, and a dense‐tooth comb, all of which are cost‐effective, portable, easily sterilized, and can be placed in the operating room. In addition, this method does not require extra chemicals or chemical modifications to the raw materials, and therefore has no adverse effects on the biological activity of sEVs. After preparing the raw materials, the entire fabrication process can be completed in 10 min or less, enabling the hydrogel to be formed for immediate use on the operating table during RCR surgery. All of these features make our new strategy for preparing macroporous hydrogels easily implementable for practical applications in a clinical setting.

The porous structure of hydrogels is essential for directing tissue repair processes such as cell adhesion, migration, infiltration, proliferation, ECM accumulation, and vascular ingrowth.^[^ ^32^ ^]^ Compared to hydrogels with submicron or nanosized pores, macroporous hydrogels with micron‐sized pores can facilitate better nutrient transportation and metabolite discharge, which may accelerate the degradation of implanted hydrogels and promote cell survival and tissue regeneration.^[^ ^29^ ^]^ In this study, we found that the macroporous hydrogels degraded much more rapidly than nanoporous hydrogels, possibly due to better solution diffusion. The alignment of microfiber gels in the macroporous hydrogels was observed to improve supraspinatus tendon repair in vivo and promote TDSC tenogenic differentiation in vitro. These findings were consistent with other studies, where aligned fibers mimicking tendon structures were more conducive to tenogenic differentiation of TDSCs and induced better tendon‐like tissue formation compared to randomly oriented fibers.^[^ ^33^ ^]^ Similarly, a multilayered aligned scaffold produced by electrospinning was reported to exhibit significantly higher Tnmd expression, more organized collagen fibrils throughout its full thickness, and higher yield stress and Young's modulus compared to a non‐aligned scaffold.^[^ ^34^ ^]^ Our previous study also showed that aligned polycaprolactone electrospun fibrous membranes could promote RCR repair.^[^ ^26^ ^]^ Studies on the mechanism by which aligned scaffold structure promotes tenogenic differentiation are limited, with one study suggesting that this was associated with integrin‐mediated signaling cascades that modulated both cell shape and intracellular signals.^[^ ^33^ ^]^ Another study demonstrated that histone deacetylases were involved in the pro‐tenogenic effects of aligned topography.^[^ ^35^ ^]^ Based on our in vitro and in vivo observations, we hypothesize that hydrogels with aligned fibers are more conducive to tenocyte recruitment in an aligned pattern in vivo and maintenance of tenocyte function, which results in the deposition of aligned collagen fibers and ECM remodeling to mimic native tendon structure. There may also be a biomechanical contribution, whereby hydrogels with aligned structures provide better mechanical support in the direction of physiological stress during tendon healing compared to hydrogels with random structure, as shown for fibrous tendon scaffolds in other studies.^[^ ^36^ ^]^ However, the detailed molecular mechanisms remain unclear and warrant further investigation.

Small extracellular vesicles from stem cells have been widely reported to have beneficial effects on cell activities, including tenogenesis.^[^ ^37^ ^]^ In this study, sEVs derived from human ADSCs were seen to enhance cell infiltration into hydrogels incorporating the sEVs compared to those that did not, suggesting that the sEVs could effectively attract stem cell migration. Nevertheless, hydrogels with aligned and random arrangements of microfiber gels showed no difference in cell infiltration. A possible reason is that these two types of hydrogels both had large microscale pores formed by the voids between microfiber gels, and different arrangements of the voids did not have a significant effect on cell infiltration through the hydrogel. Similarly, there was no difference in the release behavior of sEVs from hydrogels with aligned or random structures, presumably due to the large pores within the hydrogel compared to the nanoscale size of the sEVs. While the sEVs induced the migration of endogenous cells into the hydrogel, the large pores of the hydrogel facilitated quick degradation that further enhanced cell infiltration, which together promoted hydrogel integration with the host tissue. The hydrogels that incorporated sEVs also showed better ability to enhance in vivo tendon repair and stimulate in vitro tenogenic differentiation of TDSCs. This is possibly due to the effect of sEVs in attenuating early host inflammatory response to implanted materials, resulting in improved tendon regeneration.^[^ ^38^ ^]^ A recent report suggests that the mechanism by which sEVs induce tenogenic differentiation through their molecular cargo was associated with SMAD signaling pathways.^[^ ^39^ ^]^

Combined structural and biochemical signals provided by biomaterials may synergistically improve cellular communication, differentiation, and tissue regeneration.^[^ ^40^ ^]^ For instance, our previous study found that aligned electrospun nanofibers and bioactive ions from bioglass jointly stimulated fibroblast–endothelial interactions to enhance chronic wound healing.^[^ ^41^ ^]^ The bioglass ionic products primarily facilitated cell–cell communication through paracrine effects, while the aligned nanofibers synergistically stimulated cell‐cell interactions through gap junctional communication. In the current study, MHA‐sEVs combining structural cues from aligned microfiber gels and biochemical cues from sEVs showed the best efficacy in osteoporotic RCR among all hydrogel groups, suggesting that the two types of cues exerted some synergistic effects. Interestingly, MHA‐sEVs promoted tenogenesis to a much greater extent than MHR‐sEVs, indicating that the aligned structure was mainly responsible for pro‐tenogenic effects while sEVs were mainly responsible for cell migration and infiltration. The detailed molecular mechanisms driving these synergistic effects require further investigation. It is worth noting that the MHA‐sEVs group still showed inferior tendon repair compared to the sham group at 8 weeks, although this duration of healing time is considered a very early stage of tissue remodeling during tendon repair. Future testing conducted at 1–2 years after surgery may reveal further improvements in tendon repair.

While osteoporosis can adversely affect TBH, superior bone regeneration in the enthesis improves this condition in RCR.^[^ ^42^ ^]^ The inflammatory response in the early phase of TBH mediated by M1 Mφ is detrimental to angiogenesis and osteogenic differentiation.^[^ ^43^ ^]^ Mitochondrial dysfunction of M1 Mφ, mediated by excessive ROS production, directly contributes to an inflammatory microenvironment and also makes it difficult to repolarize M1 Mφ into anti‐inflammatory M2 Mφ.^[^ ^44^ ^]^ Promoting Mφ polarization to M2 may reverse these effects and create a regenerative microenvironment conducive to tissue repair.^[^ ^45^ ^]^ Unfortunately, clinically used anti‐inflammatory medications, such as glucocorticoids and non‐steroidal anti‐inflammatory drugs, typically relieve symptoms rather than reduce or prevent inflammatory attacks.^[^ ^46^ ^]^ ADSCs are known to exert immunomodulatory effects by secreting a myriad of growth factors and anti‐inflammatory cytokines involved in regulating the pathology of many inflammation‐related diseases.^[^ ^47^ ^]^ Moreover, ADSCs have been used in clinical trials to treat rotator cuff disease, which showed no adverse effects while improving shoulder function and reducing pain.^[^ ^48^ ^]^ Similar to the parent ADSCs, sEVs derived from ADSCs confer significant anti‐inflammatory effects.^[^ ^49^ ^]^ The sEVs also present distinct advantages compared to ADSCs, such as minimal immunogenicity even when applied xenogenetically and the ability to be used as an off‐the‐shelf therapy.^[^ ^50^ ^]^ sEVs therefore exhibit significant potential to be used as bioactive factors to replace or augment current anti‐inflammatory drugs in tendon repair.

Based on the progression of natural tendon repair, in this study, we examined inflammatory indicators at the TBI at 2 weeks following surgery in a rat osteoporotic RCR model.^[^ ^51^ ^]^ The hydrogels incorporating sEVs could provide sustained release over two weeks, allowing the sEVs to participate in immunomodulation during the inflammation phase of tendon repair. Our investigations suggested that sEVs were the primary contributor to the anti‐inflammatory functions of sEVs‐incorporated hydrogels, such as promoting M2 Mφ polarization, increasing IL‐10 secretion, and reducing TNF‐α secretion. Nevertheless, part of the hydrogel's anti‐inflammatory effects might be attributed to the inclusion of HA in its composition, as well as aligned structure in the groups comprising aligned microfiber gels.^[^ ^52^ ^]^ To this end, a previous study has shown that aligned nanofibers/microfibers could better attenuate inflammation compared to non‐aligned fibers by modulating Mφ polarization.^[^ ^53^ ^]^

Limited studies have revealed the mechanisms by which sEVs from ADSCs promote M0 Mφ polarization to M2, with one report indicating associations with the miR‐451a/macrophage migration inhibitory factor (MIF) pathway in bone regeneration.^[^ ^22^ ^]^ However, this study only performed microarray and bioinformatics analysis of sEVs, without identifying the gene expression changes of Mφ after sEVs treatment. In the present study, we found obvious upregulation of the NF‐κb signaling pathway in Mφ stimulated by LPS treatment, which was significantly down‐regulated after adding sEVs. Suppressing the NF‐κb signaling pathway has been linked to promoting Mφ phenotype conversion from M1 to M2.^[^ ^54^ ^]^ Additionally, studies have shown that this pathway is activated in clinical rotator cuff tendinopathy.^[^ ^55^ ^]^ In preclinical studies, IKKβ conditional knockout mice, which cannot activate NF‐κb signaling showed accelerated TBH after rotator cuff injury, while IKKβ activation delayed TBH.^[^ ^56^ ^]^ Given the key role of NF‐κb signaling in Mφ polarization and TBH, combined with the current RNA‐sequencing results, we focused on the changes in NF‐κb signaling in M1 Mφ after the addition of sEVs. In this study, the sEVs effectively improved mitochondrial dysfunction in M1 Mφ and prevented Mφ conversion to M1 through the NF‐κb signaling pathway. In addition, the sEVs promoted Mφ polarization to M2, which helped to create a regenerative microenvironment for RCR.

Improving the inflammatory microenvironment can create favorable conditions for both vascular and bone regeneration during the proliferative and remodeling phases of tissue healing.^[^ ^57^ ^]^ Neovascularization is required to supply nutrients and transport calcium, phosphorus, and other minerals to the defect area to facilitate bone regeneration.^[^ ^58^ ^]^ Rotator cuff tears reduce the blood supply to the TBI, which can adversely affect the healing of the postoperative bone tunnel.^[^ ^59^ ^]^ In this study, MHA‐sEVs displayed the best vascular regeneration effect at 4 weeks and bone repair performance at 8 weeks among all groups. Interestingly, at 8 weeks after surgery, MHA‐sEVs showed lower angiogenesis compared to the other hydrogel groups and control, possibly because tissue repair has entered the remodeling phase at this point when a reduction in the number of vessels decreases the incidence of tissue fibrosis.^[^ ^60^ ^]^ In vitro experiments also confirmed that treatment with sEVs largely reversed the inhibitory effect of the M1 Mφ conditioned medium on osteogenic differentiation of BMSCs and vascularization of HUVECs. These results collectively pointed to the trophic functions of sEVs that can dampen the inflammatory effects of M1 Mφ following injury, thereby creating a more regenerative microenvironment for TBH in osteoporosis. However, specific substances such as miRNAs and proteins in the sEVs that underlie their biological function in modulating inflammatory responses need further investigation. While M1 Mφ are the dominate population during the early stage of tendon‐bone healing, sEVs may similarly have a positive effect on enhancing the activity of M2 Mφ in the later repair stages.^[^ ^61^ ^]^ The influence of sEVs on M2 Mφ in the context of vascular and bone regeneration using our hydrogel system would be interesting to explore in future studies.

The tendon and bone regions in the native TBI are bridged by an interfacial fibrocartilaginous zone, comprising nonmineralized fibrocartilage adjacent to the tendon and mineralized fibrocartilage adjacent to the bone.^[^ ^62^ ^]^ Tendon and bone have different tensile moduli and the fibrocartilaginous zone allows better stress transfer between these two tissue types at the TBI.^[^ ^63^ ^]^ Conventionally, the rotator cuff heals by scarring after surgical repair. Since fibrous scar tissue is less capable of transmitting load and dispersing stress than fibrocartilage, reconstruction of the fibrocartilaginous zone within the TBI is imperative for reducing the chance of retear following RCR.^[^ ^64^ ^]^ In this study, MHA‐sEVs showed the most prominent fibrocartilaginous regeneration among all groups. The mechanism might be explained by the findings of our previous study, showing that modulation of the Mφ‐mediated inflammatory response enhanced chondrogenic differentiation in BMSCs, which in turn contributed to cartilage repair.^[^ ^65^ ^]^ Successful reconstruction of the TBI is essential for improving the mechanical strength of the supraspinatus‐humerus complex.^[^ ^61^ ^]^ Compared with other groups, MHA‐sEVs were the most effective at improving the biomechanics of the tendon‐to‐bone complex, which might reflect the better ability of this hydrogel in promoting TBH and reducing the chance of retear. In addition, this study used a model of acute rotator cuff injury, but our future research will focus on promoting tendon‐bone healing in chronic rotator cuff injuries since these are more prevalent in clinical practice.

## 4 Conclusion

This study is the first report of a macroporous hydrogel that simultaneously provides an aligned structure and immunomodulatory effects conferred through sEVs for enhancing TBH. Dual effects are seen whereby the hydrogel's macroporous and aligned structure enhances cell infiltration and tenogenic differentiation of TDSCs, acting synergistically with the release of embedded sEVs to modulate the inflammatory microenvironment in tendon injury and enhance vascularized bone regeneration. In an osteoporotic RCR model, the hydrogel improved fibrocartilage formation at the rotator cuff TBI and enhanced the biomechanical strength of the regenerated supraspinatus‐humerus complex. The sEVs acted by ameliorating M1 Mφ mitochondrial dysfunction and inhibiting Mφ polarization toward M1 through the NF‐κb signaling pathway, which effectively reduced the secretion of inflammatory factors by Mφ. The hydrogel developed in this study features a simple and efficient preparation method, which, together with its multifaceted regenerative functions, is anticipated to provide an effective means of promoting RCR in clinical practice.

## Conflict of Interest

The authors declare no conflict of interest.

## Supporting information

Supporting Information

## Data Availability

The data that support the findings of this study are available from the corresponding author upon reasonable request.
