# Supplementary material for: Macroporous Granular Hydrogels Functionalized with Aligned Architecture and Small Extracellular Vesicles Stimulate Osteoporotic Tendon‐To‐Bone Healing
Source: Adv Sci (Weinh). 2023 Oct 22;10(34):2304090. doi: 10.1002/advs.202304090 (PMC10700691; doi:10.1002/advs.202304090)
Supplement: Supplementary file 1 — Supporting Information [file ADVS-10-2304090-s001.pdf]

ADVANCED SCIENCE
Open Access

Supporting Information

for *Adv. Sci.*, DOI 10.1002/advs.202304090

Macroporous Granular Hydrogels Functionalized with Aligned Architecture and Small Extracellular Vesicles Stimulate Osteoporotic Tendon-To-Bone Healing

Wei Song, Zhijie Ma, Xin Wang, Yifei Wang, Di Wu, Chongyang Wang, Dan He, Lingzhi Kong, Weilin Yu, Jiao Jiao Li, Haiyan Li and Yaohua He**

Supporting Information for

Macroporous Granular Hydrogels Functionalized with Aligned Architecture and Small Extracellular Vesicles Stimulate Osteoporotic Tendon-to-Bone Healing

Wei Song, Zhijie Ma, Xin Wang, Yifei Wang, Di Wu, Chongyang Wang, Dan He, Lingzhi Kong, Weilin Yu, Jiao Jiao Li, Haiyan Li, Yaohua He**

1. Experimental Section

1.1 Cell isolation and culture

The murine macrophage (Mφ) RAW 264.7 cell line was purchased from the cell bank of the Chinese Academy of Sciences (Shanghai, China). Human umbilical vein endothelial cells (HUVECs) and human adipose tissue mesenchymal stem cells (ADSCs) were obtained from Zhong Qiao Xin Zhou Biotechnology Co. The isolation of human bone marrow-derived mesenchymal stem cells (BMSCs) and human tendon-derived stem cells (TDSCs) was approved by the Ethics Committee of the Shanghai Sixth People's Hospital (2019-KY-036(K), 2023-KY-074(K)). BMSCs were extracted from femoral bone marrow collected from patients who underwent total hip arthroplasty, according to published methods.[1] TDSCs were isolated from trimmed hamstring tendons during anterior cruciate ligament reconstruction. The tendons were trimmed into small pieces using microscopic scissors and digested with 2 mg/mL collagenase for 4 h. The digested tendon tissue was then placed in a 6-cm diameter culture dish, and culture medium was added. Half of the culture medium was changed after 3 days, and all medium was changed after 5 days. Tendon outgrowth primary cells were digested with trypsin and passaged when they reached 80-90% confluence, while cells at passage 3 were used as TDSCs. ADSCs, BMSCs, TDSCs, and Mφ were cultured in Minimum

Essential Medium α (α-MEM) containing 10% fetal bovine serum (FBS; Gibco, USA) and 1% penicillin/streptomycin (Hyclone, USA). HUVECs were cultured in endothelial cell medium (Sciencell, USA).

1.2 Identification of ADSCs

Trilineage differentiation in ADSCs at passage 3 was assessed by osteogenic, chondrogenic, and adipogenic assays. For osteogenesis, ADSCs were seeded at 4×10^4 cells/well in 24-well plates, and osteogenic differentiation medium (Cyagen, China) was added after the cells reached 80% confluence. The medium was changed every 3 days, and Alizarin red (Solarbio, China) staining was performed after 3 weeks. For chondrogenesis, 4×10^5 ADSCs were transferred to 15 mL centrifuge tubes and centrifuged at 400 g. Chondrogenic differentiation medium (Cyagen, China) was added to the tube and changed every 3 days. After 3 weeks, chondrogenic pellets were frozen with optimal cutting temperature compound (OCT; Solarbio, China) and cryo-sectioned, and the sections were stained with Alcian blue (Solarbio, China). For adipogenesis, ADSCs were seeded at 4×10^4 cells/well in 24-well plates. After the ADSCs reached 100% confluence, adipogenic differentiation medium A (Cyagen, China) was added for 3 days, followed by addition of adipogenic differentiation medium B for 1 day. After 4 times of alternating incubation in mediums A and B, the cells were maintained in medium B for 4-7 days until lipid droplets became large and round. Oil red O (Cyagen, China) staining was then performed.

For flow cytometry, ADSCs were prepared as cell suspensions and added to 1.5 mL EP tubes (1×10^6 cells/tube). Primary antibodies were incubated at 4 °C for 30 min and protected from light. The antibodies APC-anti-human CD29, APC-anti-human CD34, PE-anti-human CD44, APC-anti-human CD90, and FITC-anti-human CD105 were respectively added. Cells were centrifuged at 1000 rpm for 5 min. Supernatants were discarded, and cells were washed repeatedly with phosphate-buffered saline (PBS). ADSCs were tested using a CytoFlex flow cytometer (Beckman Coulter, Inc., USA). The number of cells detected was 10,000. Antibodies for flow cytometry are described in Table S1. Data analysis was performed using kaluza analysis software.

1.3 Isolation of ADSCs-derived small extracellular vesicles (sEVs)

The sEVs were isolated by differential ultracentrifugation following the 2018 International Society for Extracellular Vesicles guidelines (MISEV2018, PMID 30637094). When ADSCs reached 80-90% confluence, the culture medium was removed and replaced with serum-free α-MEM medium. ADSCs were incubated in serum-free medium for 48 h, and the supernatant was collected and centrifuged at 300 g for 10 min, 2,000 g for 20 min, and 10,000 g for 30 min, followed by filtering through a 0.22 μm Sterilize Steritop filter (Millipore) to remove cells, cell debris and microvesicles. The supernatant was then ultracentrifuged twice at 1×10^5 g for 70 min to obtain sEVs. The supernatant was removed, and the sEVs pellet was resuspended in 200 μL PBS.

1.4 Characteristics and internalization of sEVs

The isolated sEVs were stained with phosphotungstic acid and photographed using 120 kV transmission electron microscopy (TEM) (Thermofisher, USA). The particle size and concentration were measured using a nanoflow cytometer (N30 Nanoflow Analyzer, Nano FCM, Inc., Xiamen, China). Surface proteins, including CD9, Tsg101, HSP70, and calnexin, were detected by western blot. Antibodies for western blot are described in Table S1-S2. Protein bands were detected with electrochemiluminescence (ECL) solution and visualized using a chemiluminescence system (Tanon-5200, China). The grayscale values of protein bands were semi-quantified with Image J software. The sEVs were labeled with PKH26 red fluorescent cell linker mini kit (Sigma-Aldrich, USA). The unbound dye was removed by centrifugation at 10^5 g. PKH26-labeled sEVs were incubated with Mφ, BMSCs and HUVECs for 48 h. Cells were fixed in 4% paraformaldehyde (PFA) and washed 3 times with PBS, followed by staining with phalloidin (1:200, Yeasen, China) for 30 min at room temperature. After 3 washes with PBS, 4′,6-diamidino-2-phenylindole (DAPI, Solarbio) was added to stain cells for 5 min. Images were taken with a DMi8 microscope (Leica, Germany).

1.5 Fabrication and characterization of macroporous hydrogels functionalized with aligned architecture and sEVs (MHA-sEVs)

1.5.1 Fabrication of the hydrogels

Sodium alginate (SA, medium viscosity) was purchased from Sigma (USA).

Methacrylated hyaluronic acid (HA) was synthesized according to published methods.[2] MHA-sEVs macroporous granular hydrogel was synthesized according to our previously reported method.[3] Briefly, SA/HA (1%/1%) solution was first prepared by dissolving SA and HA in double-distilled water (ddH_2O). Then, SA/HA was mixed with photoinitiator LAP (Sigma, USA) at a ratio of 9:1. sEVs were added to the SA/HA solution at a concentration of 1×10^{10}/mL to form a SA/HA-sEVs hydrogel precursor. SA/HA-sEVs microfiber gels were formed by wet spinning the SA/HA-sEVs hydrogel precursor in a 1 mL syringe through a 27G needle into 0.2 M $CaCl_2$ solution, where Ca^{2+} causes the gelation of SA macromolecular chains while HA macromolecules and sEVs are wrapped in the SA microfibers. To remove excess Ca^{2+}, the SA/HA-sEVs microfiber gels were washed by ddH_2O. The microfiber gels were then transferred into rectangular molds and oriented in a certain direction with a dense-toothed comb. The photoinitiator LAP was activated with UV light (365 nm, Scientz Biotechnology Co., Ltd, China) for 20 s to generate free radicals. The free HA macromolecular chains were further crosslinked to form more stable MHA-sEVs by free radical polymerization. To synthesize macroporous granular hydrogels with random microfiber gels and sEVs (MHR-sEVs), the same steps were performed as above for MHA-sEVs, except the microfiber direction was not combed. Additionally, macroporous granular hydrogels with aligned microfiber gels only (MHA) and macroporous granular hydrogels with random microfiber gels only (MHR) were synthesized as above, but without the addition of sEVs. Nanoporous SA/HA hydrogels (NH) were synthesized by mixing the SA/HA solution with photoinitiator LAP at a ratio of 9:1 and injecting the solution directly into the mold, followed by UV crosslinking. To facilitate observation of the microfiber gels, FITC (Thermo Fisher, USA) was added to the SA/HA solution, and the microfiber gels were observed and photographed using a fluorescence microscope (Leica DMI 3000B, Germany).

1.5.2 Characterization of the hydrogels

The surface morphology of hydrogels was examined using a scanning electron microscope (Hitachi S-4800, Japan) after freeze-drying and sputter coating, and hydrogel cross-sections were also observed after cutting the hydrogels with a surgical

blade.

The sEVs release assay was performed as follows: 500 μL of SA/HA-sEVs solution was used to fabricate MHR-sEVs and MHA-sEVs. The hydrogels were immersed in 1 mL PBS. At predetermined time points (3 h, 8 h, 1 day, 2 days, 3 days, 5 days, 7 days, 10 days, 14 days), 200 μL of supernatant was collected, and the same volume of fresh PBS was added. The protein content of the collected supernatant was determined using the enhanced BCA protein assay kit (Beyotime, China). The amount of sEVs released was determined by measuring the protein content in the collected supernatant. The cumulative sEVs release rate was calculated for MHR-sEVs and MHA-sEVs.

The hydrogel degradation experiments were performed as follows: SA/HA (1mL) or SA/HA-sEVs solution (1mL) was used to fabricate NH, MHR, MHA, MHR-sEVs and MHA-sEVs. Different hydrogels were immersed in 5 mL of PBS at 37 °C. At predetermined time points (0 h, 8 h, 1 day, 2 days, 3 days, 5 days, 7 days, 10 days, 14 days, 21 days), the remaining hydrogels were removed from the immersion medium and gently washed with ddH_2O. After removing water from the surface, the hydrogels were weighed, and the weight change of the hydrogel was calculated as the hydrogel mass at each time point divided by the initial hydrogel mass.

1.6 In vitro biocompatibility assessment of MHA-sEVs

To evaluate hydrogel biocompatibility, extracts of different hydrogels were used to culture cells. The hydrogel extracts were prepared as follows: hydrogels were placed in α-MEM at 0.1 g/mL and incubated for 72 h at 37 °C. The extracts were filtered through a 0.22 μm filter and stored at 4 °C. Before culturing cells, complete medium was prepared by adding 10% FBS and 1% penicillin/streptomycin to the extracts. For HUVECs treatment, endothelial cell medium was used to prepare extracts. Then, Mφ, BMSCs, and HUVECs were seeded into 96-well plates at 1×10^3 cells/well. Cells were treated with complete medium or extracts of MHR, MHA, MHR-sEVs, and MHA-sEVs. At the scheduled time, cells were incubated in complete medium containing 10% Cell Counting Kit-8 (CCK-8, Beyotime, China) solution for 2 h. The absorbance of each group was obtained at 450 nm using a microplate reader (BioTek Inc., USA). The Calcein/PI Cell Viability Assay Kit (Beyotime, China) was used to assess the

biocompatibility of hydrogels. Cells were seeded in 96-well plates using the same procedure as for the CCK-8 experiment. Calcein AM (green fluorescence)/PI (red fluorescence) assay working solution was added and incubated for 30 min at 37 °C. Images were taken with a DMi8 microscope.

1.7 Subcutaneous implantation of MHA-sEVs

The subcutaneous implantation experiment was approved by the Institutional Animal Care and Use Committee of Shanghai Sixth People's Hospital (DWLL2023-0441). Thirty SD rats (250 ± 20 g) were divided into five groups (NH, MHR, MHA, MHR-sEVs, and MHA-sEVs), with 6 rats in each group. The hair on the back of the rats was shaved, and a longitudinal skin incision of 1.5 cm in length was made. After subcutaneous implantation of different hydrogel groups, specimens were collected at 2 and 4 weeks postoperation. The specimens were fixed with PFA for 24 h, followed by gradient dehydration and paraffin embedding. Hematoxylin and eosin (H&E) staining was performed after cutting into 5 μm thick paraffin sections. The relative number of infiltrated cells in the hydrogels and relative remaining volume of hydrogels were calculated using Image J software.

1.8 Osteoporotic rotator cuff repair (RCR) model in rats

The animal study was approved by the Institutional Animal Care and Use Committee of Shanghai Sixth People's Hospital (DWLL2023-0441). A total of 107 female SD rats (250 ± 20 g) were used in the study. To develop osteoporosis in the rats, bilateral ovaries were removed in 101 rats, and the remaining 6 rats without ovariectomy served as the normal group. The rats were anesthetized with 0.5% sodium pentobarbital intraperitoneally. The lateral abdominal hair of the rats was shaved, and a 1 cm incision was made into the abdominal cavity to resect the bilateral ovaries (2 rats died postoperatively). After 13 weeks, 6 of the 99 osteoporotic rats without RCR were used as the sham group. Micro-computed tomography (μ-CT) was performed in the sham group and normal rats (without ovariectomy) to determine whether osteoporosis had been successfully modeled. The remaining 93 osteoporotic rats receiving RCR were divided into 5 groups (3 died intraoperatively, 90 remaining): Control group (direct suture), MHR group (direct suture + MHR), MHA group (direct suture + MHA), MHR-

sEVs group (direct suture + MHR-sEVs), and MHA-sEVs group (direct suture + MHA-sEVs). The acute RCR model was developed according to our published methods.[4] Rats were anesthetized by 0.5% pentobarbital sodium intraperitoneally. After anesthesia, the supraspinatus tendon was severed bilaterally at the humeral insertion. A 0.5 mm diameter drill was used to make a bone tunnel in the humerus. The supraspinatus tendon was sutured through the bone tunnel to its original attachment site using a 4-0 suture (Jinhuan Medical, Shanghai, China). After suturing, different hydrogel groups were applied to cover the surface of the tendon-to-bone interface, and the deltoid muscle and skin were then closed. Rats were injected with penicillin to prevent postoperative infection. Six rats in each group were euthanized by intraperitoneal administration of sodium pentobarbital overdose at 2, 4 and 8 weeks postoperation for assessment by gross morphological observation, μ-CT, histology, immunofluorescence, immunohistochemistry, biomechanical testing, and biosafety evaluation.

1.9 Micro-computed tomography (μ-CT) analysis

The supraspinatus-humerus complex was scanned using a μ-CT system (SkyScan 1176, Bruker, Germany), with scanning accuracy of 18 μm, scanning voltage of 90 kV, and scanning current of 278 μA. Images were analyzed using DataViewer and CTAn μ-CT software version 1.13 CT Analyser. The area of interest included the area between the footprint area of the supraspinatus tendon insertion and the growth plate of the humeral head. The parameters analyzed included the bone volume/total volume fraction (BV/TV), trabecular number (Tb. N), trabecular thickness (Tb. th), and trabecular separation (Tb. sp).

1.10 Histological, immunofluorescence, and immunohistochemical analyses

Supraspinatus-humerus complex specimens were fixed in PFA for 48 h and then decalcified and dehydrated in gradient alcohol. After paraffin embedding, specimens were cut into 5 μm thick sections, which were used for histology, immunofluorescence, and immunohistochemistry. Tissue sections were stained with H&E, safranin O-fast green, and picrosirius red. Image J software was used for semi-quantitative analysis of the positive areas of Safranin O-fast green staining. Images of picrosirius red staining

were taken using a polarized light microscope (Eclipse E800, Nikon). Ten regions (50×50 μm) in the tendon zone of the tendon-bone interface were selected for analysis. The images were imported into Image J software and converted to 8-bit format, and the grayscale values of the images were calculated. For semi-quantitative analysis, a higher grayscale value represents a more mature tendon.[5] Histological scoring was performed according to published methods, with specific scoring details shown in Table S4.[6]

For immunofluorescence assays, sections were incubated with primary antibody overnight. After washing with PBS, sections were incubated with the corresponding secondary antibody. Cell nuclei were stained with DAPI. The primary antibodies CD68, CD86, CD206, tumor necrosis factor-alpha (TNF-α), and interleukin-10 (IL-10) were used to detect Mφ polarization and inflammatory status at 2 weeks postoperation. CD31/α-Smooth muscle actin (α-SMA) immunofluorescent double-label staining was used to detect postoperative vascular regeneration at the tendon-bone interface. Co-staining of CD31 with α-SMA is frequently used to indicate the maturation of blood vessels.[7] The primary antibodies bone morphogenetic protein 2 (BMP2) and runt-related transcription factor 2 (Runx2) were used to assess postoperative bone regeneration. Image J software was used for semi-quantitative analysis of fluorescence staining intensity. A similar procedure was performed for immunohistochemical staining, using the primary antibodies collagen type I (COL I), COL II, and COL III. Antibodies for immunohistochemistry and immunofluorescence are listed in Table S1-S2. Images were acquired using a digital slide scanner (Pannoramic MIDI; 3DHISTECH Ltd). The sum integrated optical density (IOD) of the COL I, COL II, and COL III was semi-quantified by ImageJ Pro Plus (IPP) software.

1.11 Biomechanical test

Collected specimens of supraspinatus-humerus complex were frozen at -80 °C and thawed before testing. The biomechanical test was performed as previously described.[8] The cross-sectional area of the supraspinatus tendon was measured using a digital vernier caliper in the middle part of the tendon. The biomechanical strength of the supraspinatus tendon-bone complex was tested using the HSS-DX1000 microbial mechanics testing machine (Jinan Heng Rui Jin Testing Machine Co., Ltd., China). The

sample was preloaded at 0.1 N and then loaded until failure under uniaxial tension at a fixed speed of 10 mm/min. The ultimate load at failure was recorded. Stiffness was calculated using the load-displacement curve. Stress was calculated as: Stress = Ultimate load to failure / Cross-sectional area.

1.12 In vivo biosafety evaluation

Blood samples were collected at 8 weeks of rotator cuff repair for routine blood and biochemical tests, including neutrophil (Gran#) count, lymphocyte (Lymph#) count, monocyte (Mon#) count, white blood cell (WBC) count, hemoglobin (HGB) level, platelet (PLT) count, red blood cell (RBC) count, alanine aminotransferase (ALT), aspartate aminotransferase (AST), urea nitrogen (UN), creatinine (CREA), and uric acid (UA) levels. The major organs (heart, liver, spleen, lungs, and kidneys) of implanted animals were collected and fixed in PFA, followed by dehydration and paraffin-embedding for H&E staining. Blood and major organ samples from the sham group were used as controls.

1.13 Effects of hydrogels on tenogenic differentiation of TDSCs

Immunofluorescence assay was performed to determine the effect of hydrogels on the tenogenic differentiation of TDSCs. Cells were seeded onto the hydrogel sample in 6-well plates at 2×10^5 cells/well. Tenogenic induction medium was added, comprising low-glucose Dulbecco's Modified Eagle Medium (DMEM), 10% FBS, 1% penicillin/streptomycin, 25 μmol/L ascorbic acid, and 25 ng/mL connective tissue growth factor. After 3 days, cells were fixed in PFA for 15 min and incubated with quickblock™ blocking buffer (Beyotime, China) for immunofluorescence staining for 15 min. Primary antibody was added and incubated overnight, including COL I, Scleraxis (Scx), and tenomodulin (Tnmd). Subsequently, the appropriate secondary antibodies (Cy3-AffiniPure Goat Anti-Rabbit IgG (H+L) or Cy3-AffiniPure Goat Anti-Mouse IgG (H+L)) were added. After washing, phalloidin staining was performed for 30 min, and DAPI staining for 5 min. Antibodies for immunofluorescence are described in Table S1-S2. Images were taken with a DMi8 microscope.

1.14 Assessing the effect of sEVs on Mφ mitochondrial dysfunction

The experiment was divided into three groups: control, LPS, and LPS+sEVs. Mφ were

seeded into 6-well plates or 24-well plates and grown for 24 h. Then, Mφ were treated with 1 μg/mL lipopolysaccharide (LPS; Sigma, USA) for 24 h in the LPS group. The LPS+sEVs group was treated with 1 μg/mL LPS and 5×10^9/mL sEVs for 24 h. The control group received no treatment. Reactive oxygen species (ROS) assay kit (Beyotime, China) was used to detect the ROS level in Mφ after sEVs treatment. After removing the culture medium, dichlorodihydrofluorescein diacetate (DCFH-DA) working solution was added to the well and incubated for 20 min at 37 °C. Cells were then washed with PBS, and the nuclei were stained with diluted Hoechst 33258 (Yeasen, China) for 15 min. MitoSOX red mitochondrial superoxide indicator (Yeasen, China) was used to detect superoxide in Mφ mitochondria after sEVs intervention. The culture medium was removed, and mitoSOX mitochondrial superoxide indicator working solution was added and incubated for 10 min at 37 °C protected from light. The nuclei were stained with Hoechst 33258 for 15 min after washing with PBS. A mitochondrial membrane potential (Δψm) assay kit with JC-1 (Beyotime, China) was used to detect the Mφ Δψm (red fluorescence due to accumulation at high ΔΨm, green fluorescence in monomer form at low ΔΨm). The JC-1 probe was diluted to a working concentration using α-MEM. Mφ were incubated for 20 min after adding the JC-1 working solution. After washing with buffer, Hoechst 33258 was added for 15 min to stain the cells. Images of the above assays were taken by DMi8 microscope.

TEM was performed to visualize Mφ. After treatment with sEVs, Mφ were transferred to 1.5 mL EP tubes. Cells were centrifuged at 1000 rpm for 5 min, 2.5% glutaraldehyde was added, and cells were fixed overnight at 4 °C. Cells were then rinsed, fixed in osmium acid, dehydrated, and embedded for ultrathin sectioning. The sections were stained with uranyl acetate and lead citrate, and dried for observation by TEM (Thermofisher, USA).

Adenosine triphosphate (ATP) assay kit (Beyotime, China) was used to detect ATP concentration in Mφ according to the manufacturer's instructions. After adding lysis solution to each well, the cells were centrifuged at 4 °C for 5 min at 12,000 g, and the supernatant was collected. ATP assay working solution was added at 100 μL per well and left at room temperature for 5 min, followed by adding 20 μL of sample or standard

to the wells and measuring the luminescence value using a multifunctional enzyme marker (BioTek, USA). An enhanced BCA protein assay kit was used to detect the intracellular protein concentration, and the ATP concentration was divided by the protein concentration for homogenization.

1.15 Assessing the effect of sEVs on Mφ polarization

To assess the effects of LPS and sEVs on Mφ polarization, cells were seeded into 6-well plates at 4×10^5 cells/well for 24 h. For the induction of M1 Mφ, 1 mg/mL LPS was applied for 24 h in the LPS group, while 1 μg/mL LPS and 5×10^9/mL sEVs were applied for 24 h in the LPS+sEVs group. For the induction of M2 Mφ, 20 ng/mL IL-4 was added for 48 h in the IL-4 group, while 20 ng/mL IL-4 and 5×10^9/mL sEVs were added for 48 h in the IL-4+sEVs group. Controls for both M1 and M2 polarization experiments were untreated Mφ.

Flow cytometry was performed as follows: Cells were washed twice with PBS containing 5% FBS pre-chilled at 4 °C and digested with trypsin. The cells were counted after centrifuging at 4 °C for 5 min at 1000 rpm and resuspending in PBS. Cells were divided into 1.5 mL centrifuge tubes with 20,000-40,000 cells per tube. Blocking was performed by adding CD16/CD21 blocking solution at 1 μL per tube and blocking for 30 min at 4 °C on a constant temperature shaker. CCR7 and CD206 antibodies were added and incubated for 30 min at 4 °C on a constant temperature shaker. Flow cytometry was performed using a CytoFLEX flow cytometer (Beckman Coulter, Inc., USA). Antibodies for flow cytometry test are described in Table S1.

Real-time quantitative polymerase chain reaction (RT-qPCR) was performed as follows: Total RNA was extracted from Mφ using Trizol-up (EZBioscience) and converted to complementary RNA. mRNA was then converted to complementary DNA using a 4x Reverse Transcription Master Mix (EZBioscience). RT-qPCR was performed using an Applied Biosystems 7500 real-time PCR system according to the manufacturer's protocol. mRNA relative expression was calculated using the $2^{-\Delta\Delta T}$ method and normalized to GAPDH. The primer sequences are listed in Table S3.

Western blot was performed as described above, with primary antibodies for Arginase-1 (Arg-1) and inducible nitric oxide synthase (iNOS). Immunofluorescence

staining was performed as described above, with primary antibodies for CD206 and iNOS. Images were taken by confocal laser scanning microscopy. Antibodies for western blot and immunofluorescence are described in Table S1-S2.

1.16 RNA-sequencing of sEVs-treated Mφ and validation by immunofluorescence and western blot

RNA-sequencing was used to detect differential gene expression after sEVs treatment in M1 Mφ. Trizol reagent (Invitrogen) was used to extract RNA from the Control, LPS, and LPS+sEVs groups. Three replicate samples were taken from each group. Transcriptome sequencing and analysis were conducted by OE Biotech, Inc. (Shanghai, China). Gene expression with fold change >2 and p-value less than 0.05 was considered valid. Oebiotech cloud platform (https://cloud.oebiotech.cn/task/) was used to process data, including differentially expressed genes (DEGs), gene ontology (GO) analysis, kyoto encyclopedia of genes and genomes (KEGG) analysis, and gene set enrichment analysis (GSEA). Immunofluorescence detection of nuclear factor-kappa B (NF-κb) p65 translocation into the nucleus was performed. The expression of NF-κb pathway-related proteins (p-IκBα, IκBα, p-P65, and P65) was detected by western blot. Antibodies used in the western blot and immunofluorescence are described in Table S1-S2. The grayscale values of the protein bands and fluorescence intensity were semi-quantified with Image J software.

1.17 Effect of sEVs on osteogenic differentiation of BMSCs in Mφ conditioned medium

To simulate the inflammatory state in rotator cuff repair, Mφ conditioned medium was collected. M0 Mφ were seeded in 6-well plates, added with 1 μg/mL LPS, and incubated for 24 h to obtain M1 Mφ. After incubating in complete medium with or without 5×10^9 sEVs for 2 days, the supernatant was collected. The M0 Mφ supernatant was also collected. Mφ medium was prepared by mixing the supernatant with fresh α-MEM at a ratio of 1:1 (M0 Mφ supernatant, M1 Mφ supernatant, and M1 Mφ supernatant+sEVs). Mφ conditioned osteogenic media (M0 Medium, M1 Medium, and M1+sEVs Medium) were made by adding osteogenic factors (100 nM dexamethasone, 250 μM ascorbic acid, and 10 mM β-glycerophosphate) to different Mφ supernatants.

BMSCs were seeded in 24-well plates or 6-well plates. After the cells reached 80% confluence, osteogenic induction medium (Control), M0 Medium, M1 Medium, and M1+sEVs Medium were added. The culture medium was changed every 3 days. Alizarin red and alkaline phosphatase (ALP) staining were performed after 7 and 14 days, respectively. For semi-quantitative analysis of Alizarin red staining, calcium deposition was eluted with 10% (w/v) cetyplyridinium chloride (Sigma-Aldrich, USA), and the OD value was measured at 562 nm. To detect ALP activity, an ALP Assay Kit (beyotime, China) was used according to the manufacturer's instructions after lysing cells with RIPA lysis buffer (beyotime, China) and adding a portion of the sample to a 96-well plate. A standard curve was plotted, and diethanolamine (DEA) enzyme activity was calculated from the standard curve. Another part of the lysate was assayed for intracellular protein concentration using the Enhanced BCA Protein Assay Kit, and ALP activity was divided by the protein concentration for homogenization.

RT-qPCR was performed to detect the expression of osteogenesis-related genes (COL I, ALP, RUNX2, osteocalcin (OCN), and osteopontin(OPN)) after osteogenic induction for 14 days, using the procedures described above. The primer sequences are shown in Table S3. The expression of osteogenesis-related proteins (ALP, OCN, COL I) after treating BMSCs with different Mφ conditioned media for 3 days was detected by immunofluorescence using the procedures described above. Antibodies used in the immunofluorescence are summarized in Table S1-S2.

1.18 Effect of sEVs on angiogenesis of HUVECs in Mφ conditioned medium

Mφ conditioned media for HUVECs culture were prepared using a similar procedure as described above for BMSCs culture. The M0 Medium, M1 Medium, and M1+sEVs Medium were prepared by mixing different Mφ supernatants with fresh endothelial cell medium at a ratio of 1:1. HUVECs were seeded in 6-well plates at 2×10^5 cells/well and cultured for 48 h in endothelial cell medium (Control), M0 Medium, M1 Medium, and M1+sEVs Medium. After treatment with different Mφ conditioned media, HUVECs were used for subsequent studies.

Matrigel (Corning, New York, USA) was added to μ-Slide angiogenic slides (ibidi) at 10 μL/well on ice, and 10,000 cells in 50 μL of cell suspension was then added to

each well. After mixing, the cells were incubated at 37 °C for 6 h, stained with calcein (BD, USA) for 30 min, and observed using a fluorescence microscope. The total number of meshes was calculated by Image J software. Scratch wound healing assay and transwell assay were used to assess the migration ability of HUVECs. The scratch assay was performed as follows: HUVECs were seeded into a 6-well plate at 3×10^5 cells/well. After the cells reached 100% confluence, a 200 μL pipette tip was used to make a uniform, straight scratch in the middle of each well. Cells were cultured in a serum-free endothelial cell medium. At 0 and 24 h, photographs of each well were taken using a light microscope (Nikon TE2000-E, Tokyo, Japan). The transwell assay was performed as follows: HUVECs were seeded at 2×10^4 cells/well into the upper chamber of Transwell plates (Corning) with 200 μL culture medium. In the lower chamber, 500 μL complete endothelial cell medium (containing 5% FBS) was added. After 12 h, migrated cells were fixed with 4% PFA, and cells were stained with 0.1% crystal violet (Solarbio, China). Photographs were taken using a light microscope (Nikon TE2000-E, Tokyo, Japan), and the number of migrated cells was counted. The expression of angiogenesis-related proteins (VEGF, CD31) after treating HUVECs with different Mφ conditioned media for another 3 days was determined by immunofluorescence using the procedures described above. Antibodies used in the immunofluorescence are shown in Table S1-S2.

1.19 Statistical analysis

All data are presented as mean ± standard deviation (SD). Statistical analysis was conducted with GraphPad Prism 9.0 (GraphPad Software Inc, USA). At least three independent samples were used for statistical analysis in each experiment. One-way analysis of variance (ANOVA) or Student's t-test was used to assess statistical significance. Post-hoc analysis was performed using the Tukey method. Differences were considered statistically significant at $P<0.05$.

2. Figures

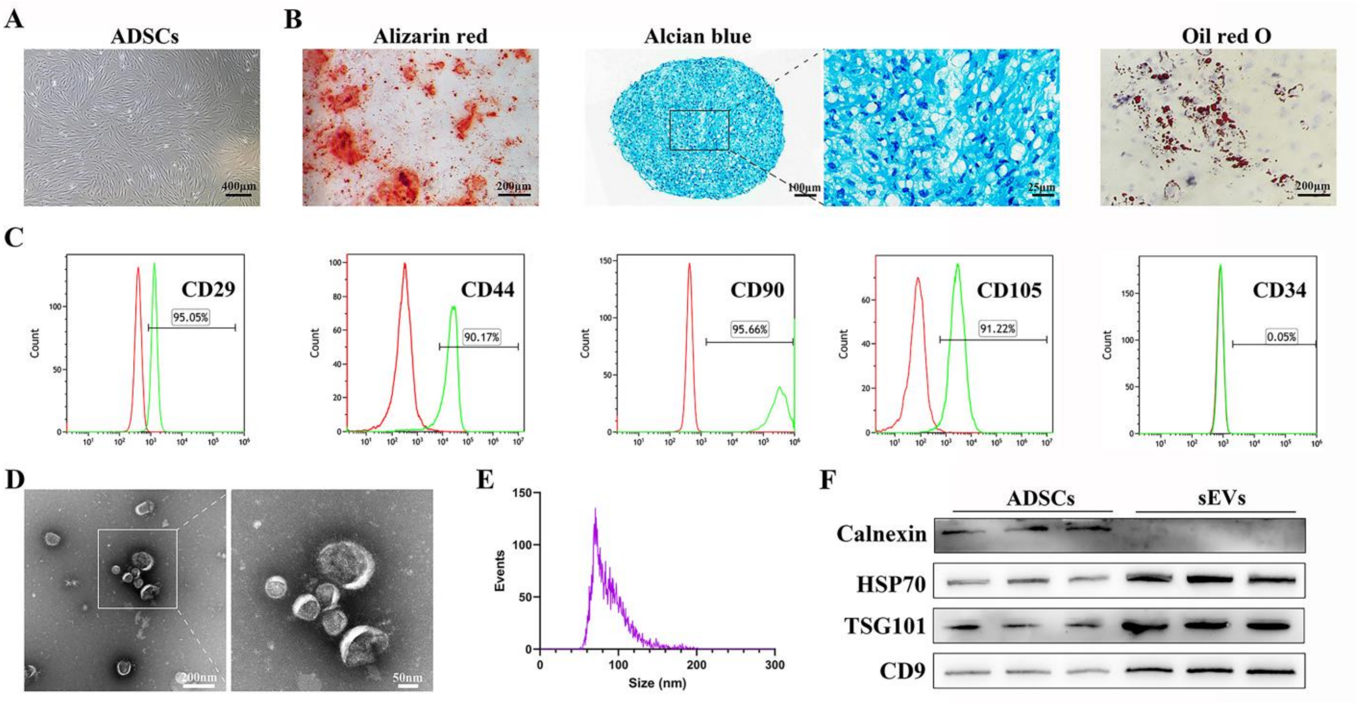

Figure S1. Identification of ADSCs and sEVs. A) ADSCs morphology shown by light microscopy. B) Representative images of Alizarin red, Alcian blue, and Oil Red O staining. C) Flow cytometry analysis of ADSCs surface markers CD29, CD44, CD90, and CD105, with CD34 as negative control. D) Morphology of sEVs observed by TEM. E) Particle size analysis of sEVs. F) Western blot analysis of Calnexin, HSP70, TSG101, and CD9 expression by ADSCs and sEVs.

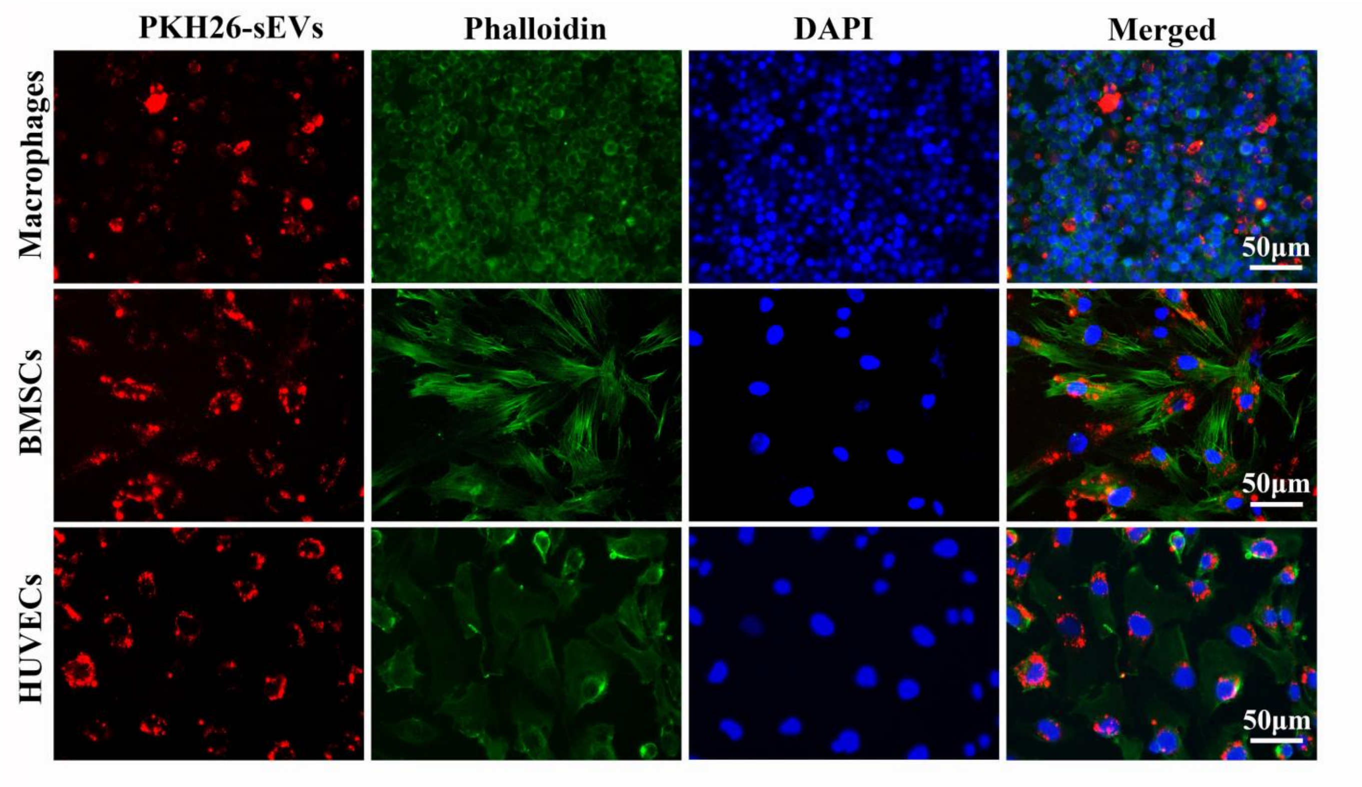

Figure S2. Uptake of PKH26-labeled sEVs by Mφ, BMSCs and HUVECs.

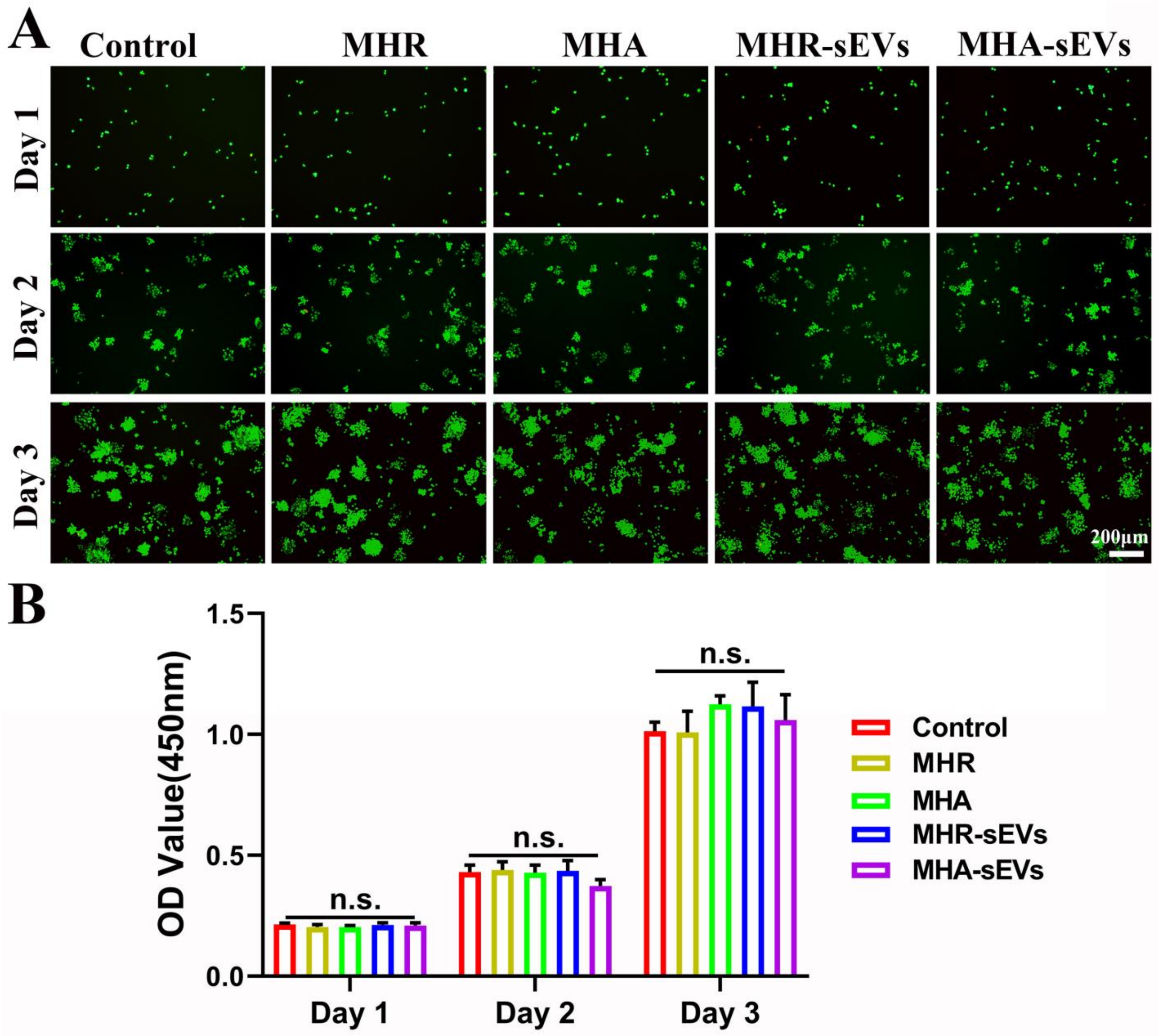

Figure S3. A) Live/dead staining of Mφ treated with different hydrogel extracts. B) Effects of different hydrogel extracts on Mφ proliferation assessed by CCK-8 assay.

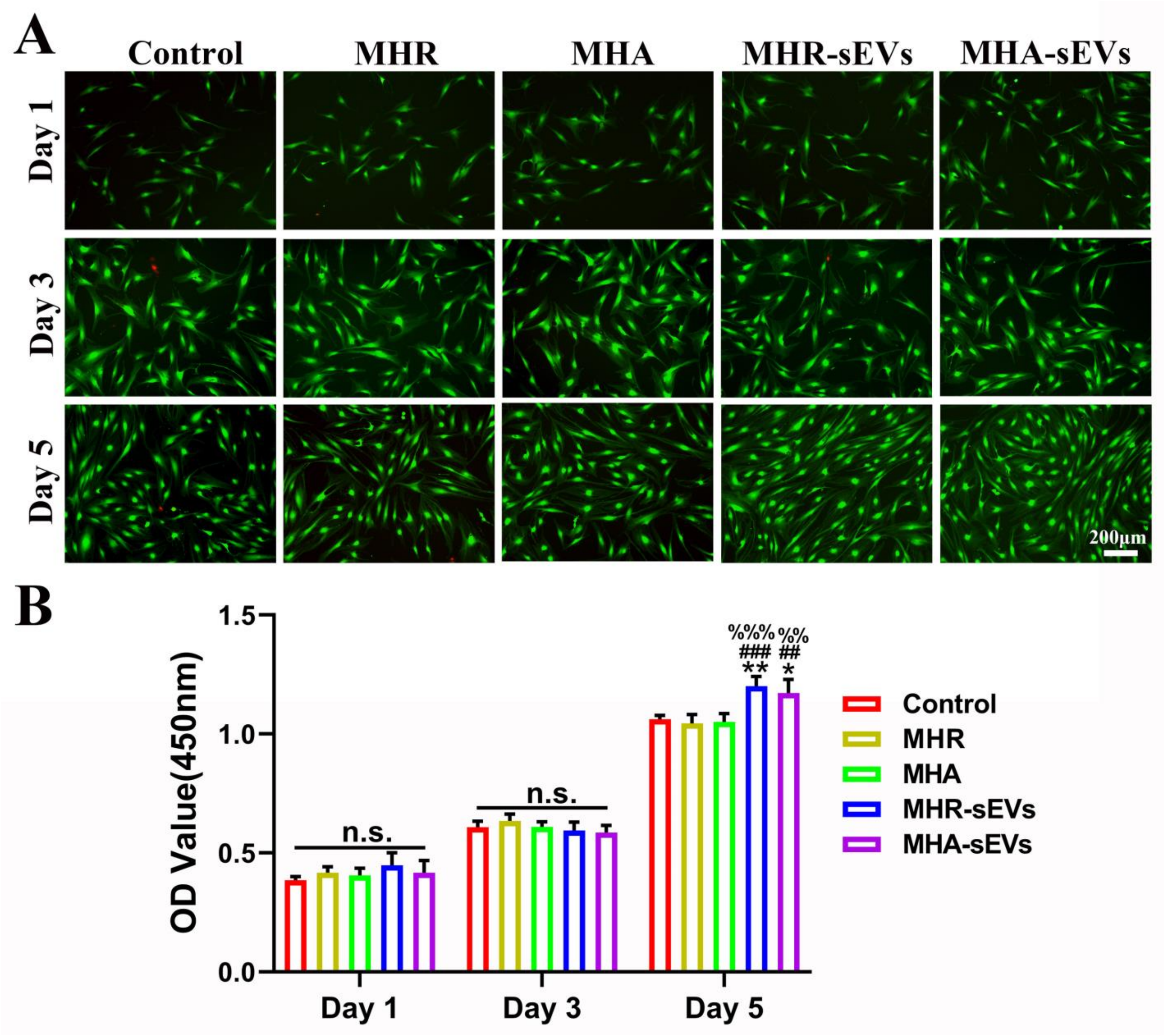

Figure S4. A) Live/dead staining of BMSCs treated with different hydrogel extracts. B) Effects of different hydrogel extracts on BMSCs proliferation assessed by CCK-8 assay. $^{*}p < 0.05$, $^{**}p < 0.01$, when the data were compared with control group. $^{\#\#}p < 0.01$, $^{\#\#\#}p < 0.001$, when the data were compared with MHR group. $^{\%\%}p < 0.01$, $^{\%\%\%}p < 0.001$, when the data were compared with MHA group.

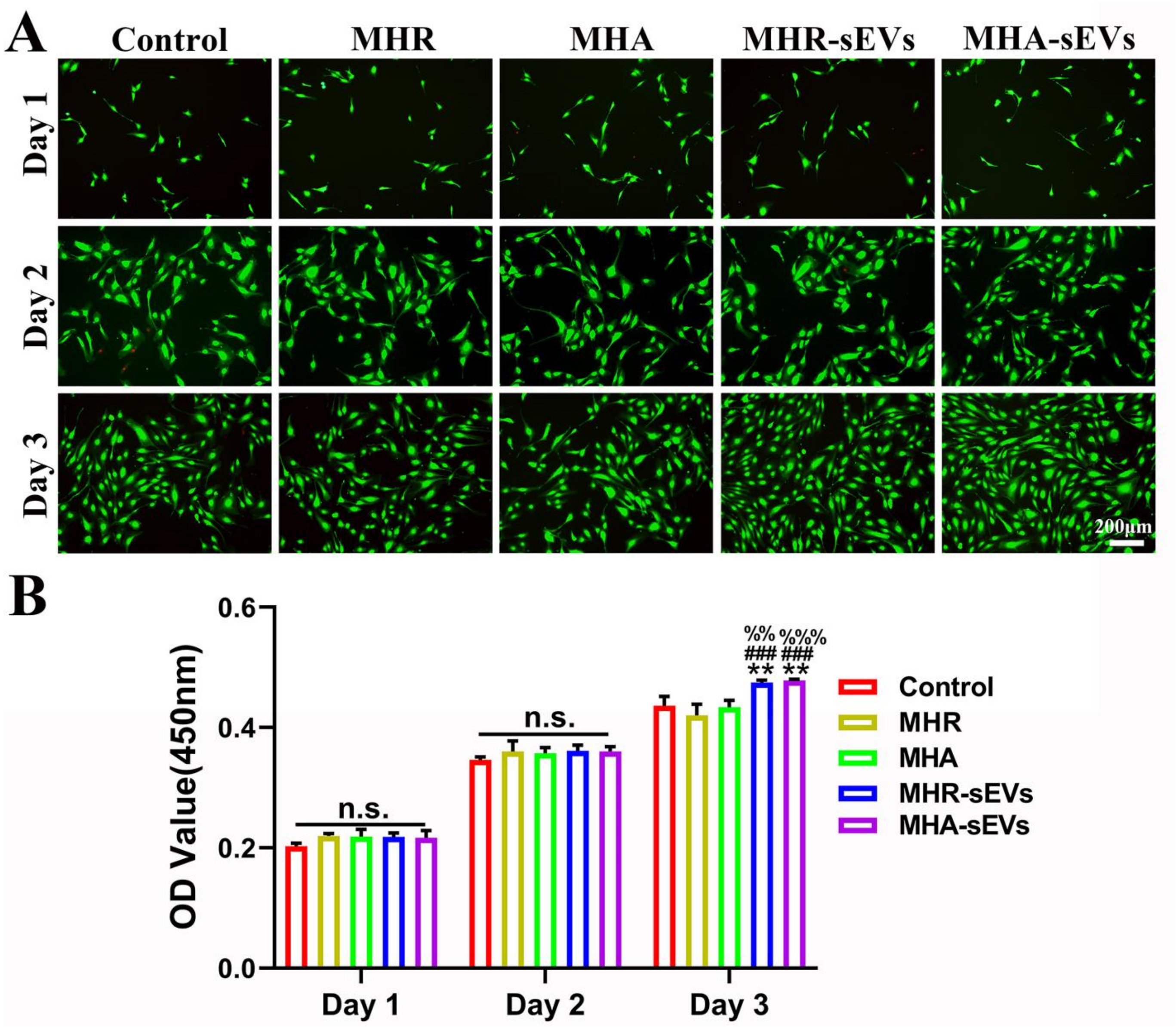

Figure S5. A) Live/dead staining of HUVECs treated with different hydrogel extracts. B) Effects of different hydrogel extracts on HUVECs proliferation assessed by CCK-8 assay. $^{**}p < 0.01$, when the data were compared with control group. $^{\#\#\#}p < 0.001$, when the data were compared with MHR group. $^{\%\%}p < 0.01$, $^{\%\%\%}p < 0.001$, when the data were compared with MHA group.

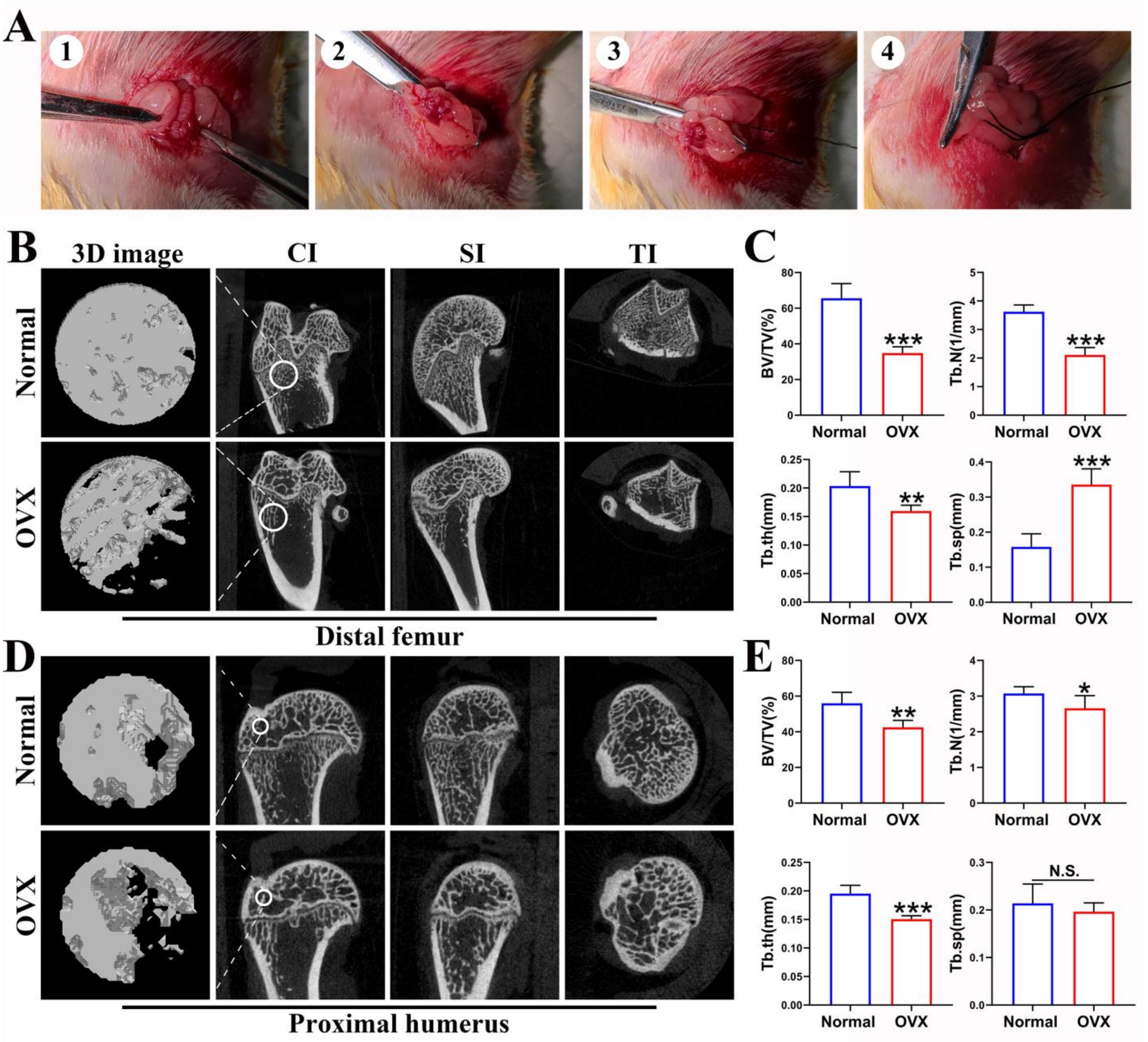

Figure S6. Establishment and confirmation of osteoporotic rat model. A) Procedure for ovariectomy: 1. locating the ovary; 2. forceps block; 3. suture ligation; 4. removal of the ovary. B) Representative 3D reconstruction, coronal, sagittal, and transverse μ-CT images of the distal femur at 13 weeks after ovariectomy. C) BV/TV, Tb. N, Tb.th, and Tb. sp of the distal femur in the normal and osteoporotic groups. D) Representative 3D reconstruction, coronal, sagittal, and transverse μ-CT images of the proximal humerus at 13 weeks after ovariectomy. E) BV/TV, Tb. N, Tb.th, and Tb. sp of the proximal humerus in the normal and osteoporotic groups. CI, coronal images; SI, sagittal images; TI, transverse images. *$P < 0.05$, **$P < 0.01$, ***$P < 0.001$, when the data were compared with Normal group.

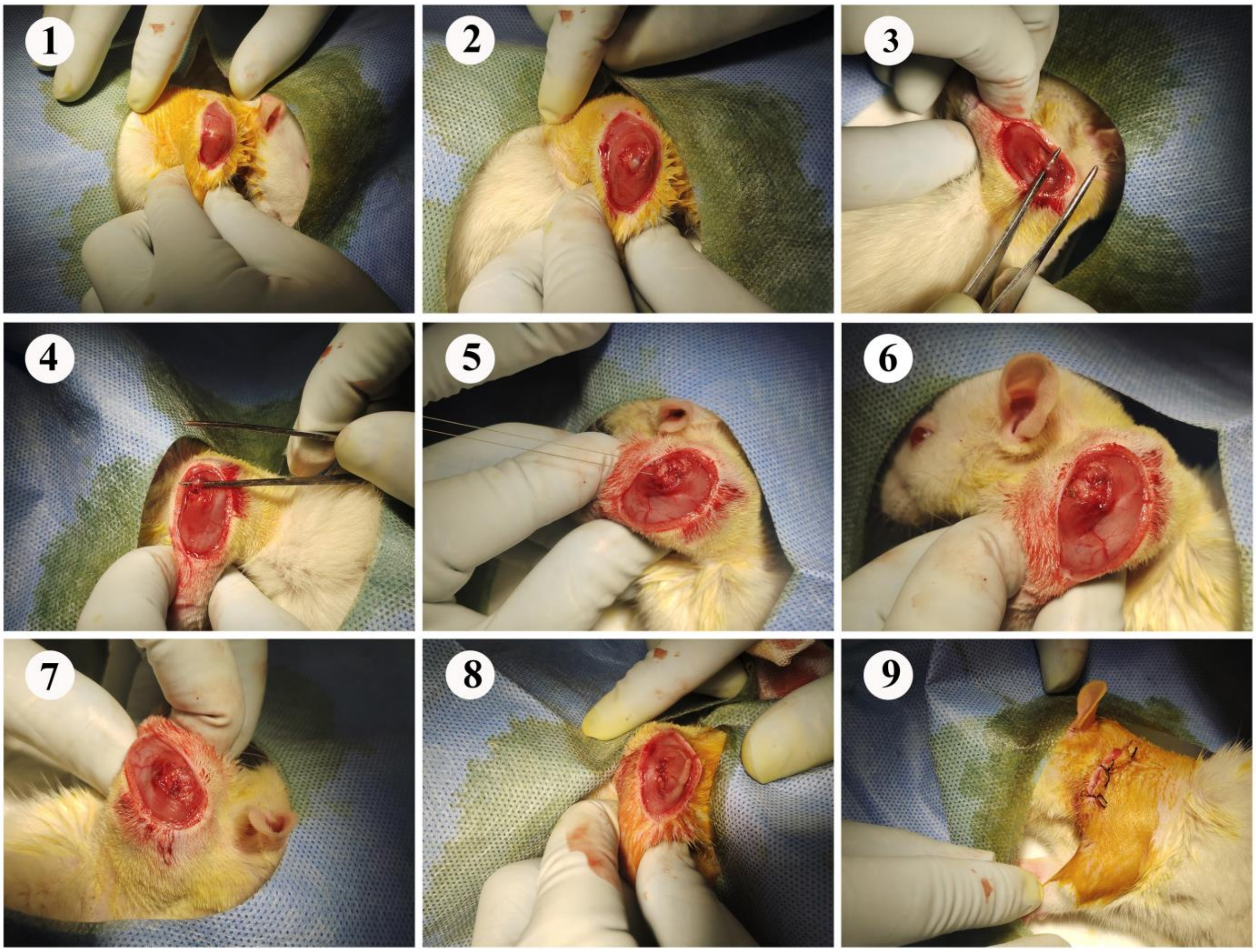

Figure S7. Establishment of a rotator cuff tear and repair model in osteoporotic rats. 1) Skin is incised; 2) The supraspinatus tendon is exposed; 3) Forceps are used to pick up the supraspinatus tendon; 4) The supraspinatus tendon is cut; 5) Suture is made through the bone tunnel; 6) The supraspinatus tendon is sutured to the humerus; 7) Hydrogel is placed; 8) The trapezius and deltoid muscles are sutured; 9) The skin is sutured.

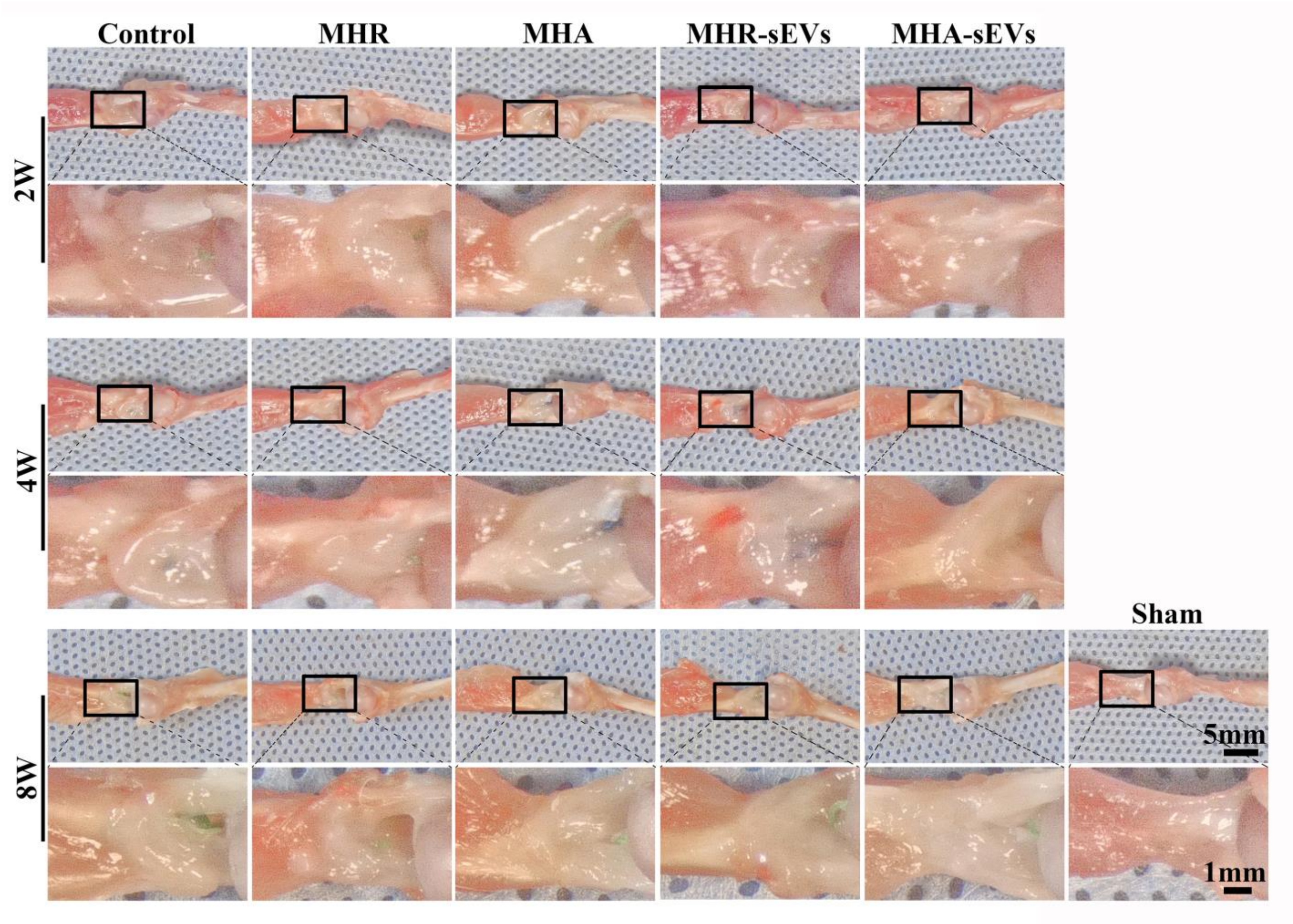

Figure S8. Gross observation of the supraspinatus-humerus complex at 2, 4, and 8 weeks of tendon repair.

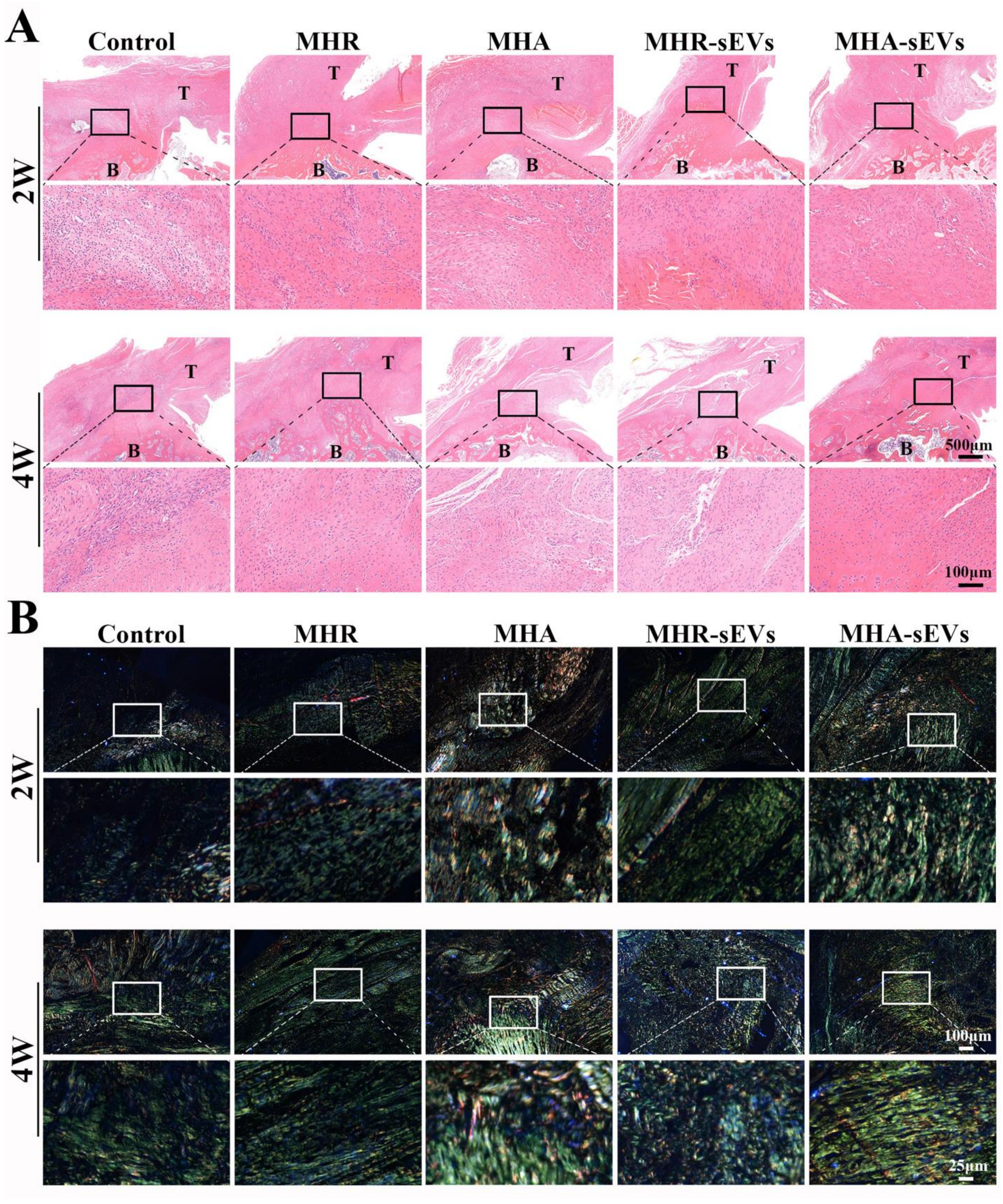

Figure S9. A) Representative H&E staining of the tendon-to-bone interface (TBI) at 2 and 4 weeks. B) Representative picrosirius red staining of the TBI at 2 and 4 weeks. B, bone; T, tendon.

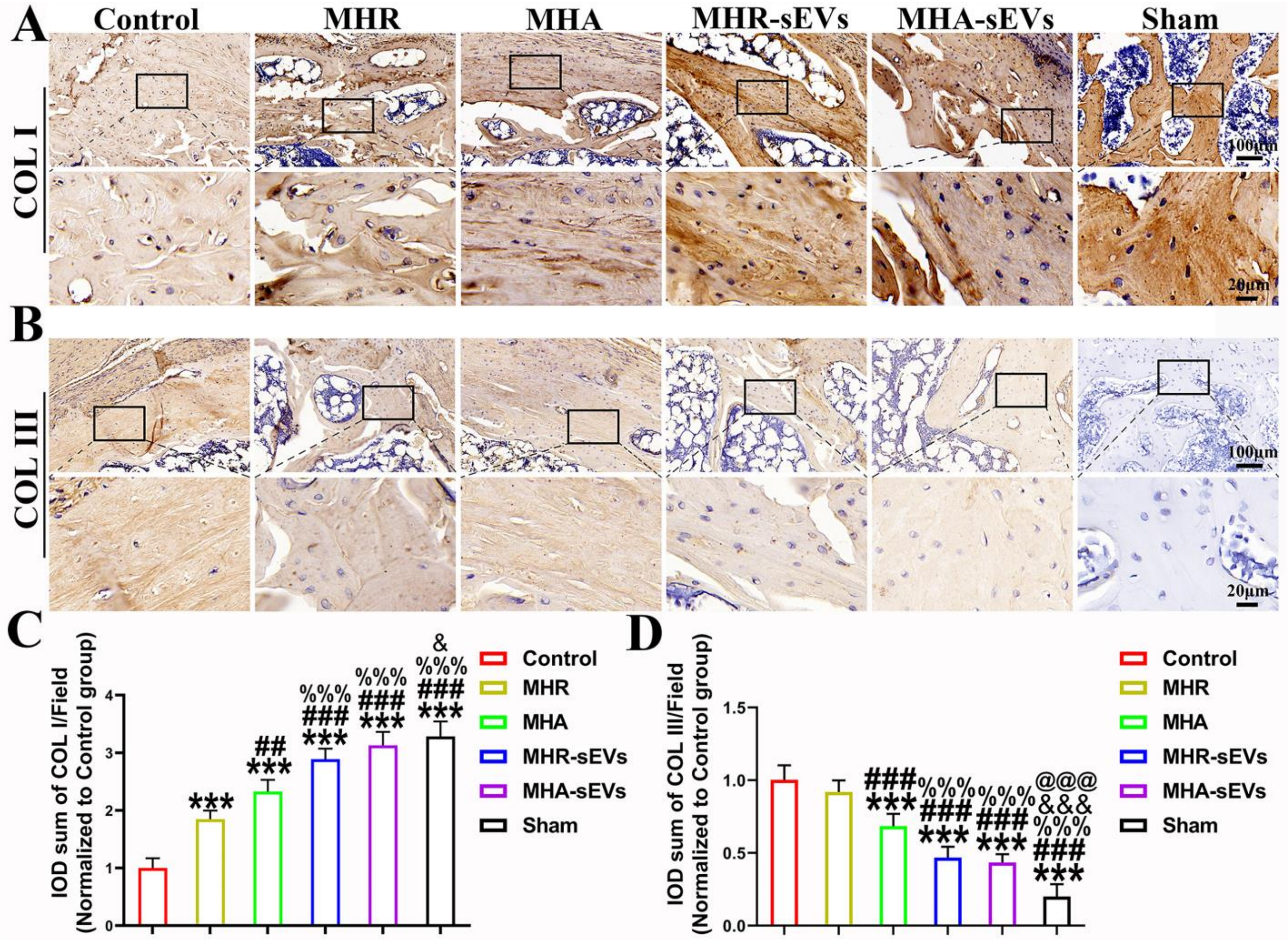

Figure S10. A) Representative immunohistochemical staining images of COL I in the bone zone. B) Representative immunohistochemical staining images of COL III in the bone zone. C) Semi-quantitative analysis of COL I. D) Semi-quantitative analysis of COL III. $^{***}p < 0.001$, when the data were compared with control group. $^{\#\#}p < 0.01$, $^{\#\#\#}p < 0.001$, when the data were compared with MHR group. $^{\%\%\%}p < 0.001$, when the data were compared with MHA group. $^{\&}p < 0.05$, $^{\&\&\&}p < 0.001$, when the data were compared with MHR-sEVs group. $^{@@@}p < 0.001$, when the data were compared with MHA-sEVs group.

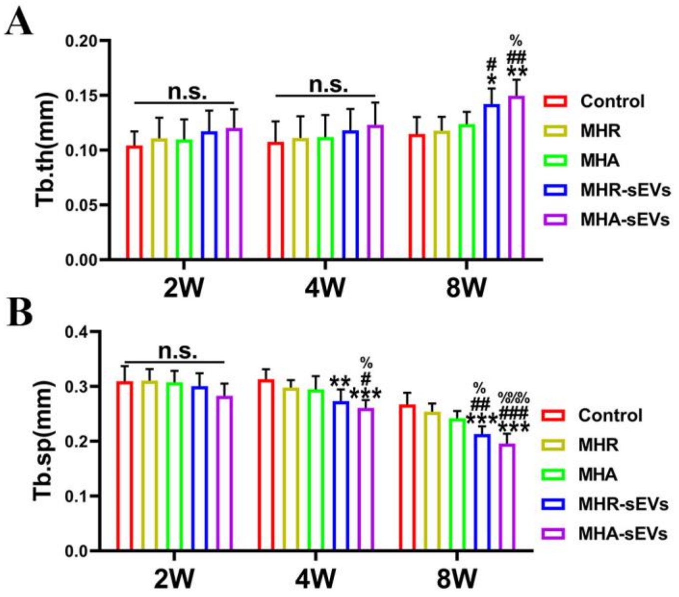

Figure S11. Semi-quantitative assessment of the regenerated bone in osteoporotic rats by μ-CT. A) Tb.th. B) Tb. sp. $^{*}p < 0.05$, $^{**}p < 0.01$, $^{***}p < 0.001$, when the data were compared with control group. $^{\#}p < 0.05$, $^{\#\#}p < 0.01$, $^{\#\#\#}p < 0.001$, when the data were compared with MHR group. $^{\%}p < 0.05$, $^{\%\%\%}p < 0.001$, when the data were compared with MHA group.

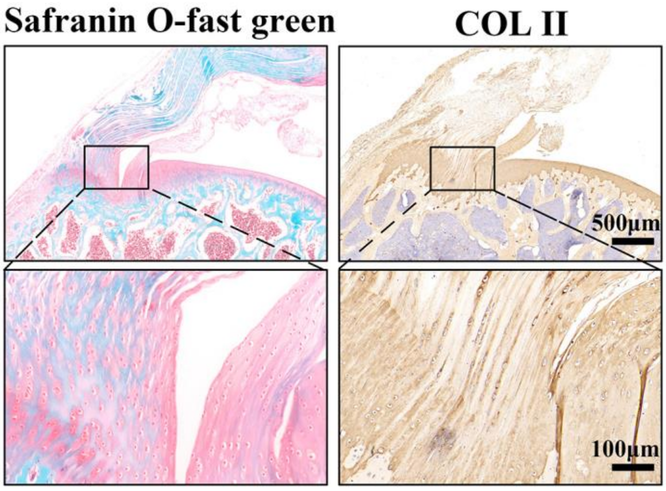

Figure S12. Representative images of Safranin O-fast green staining and COL II immunohistochemical staining images of the TBI (sham group) .

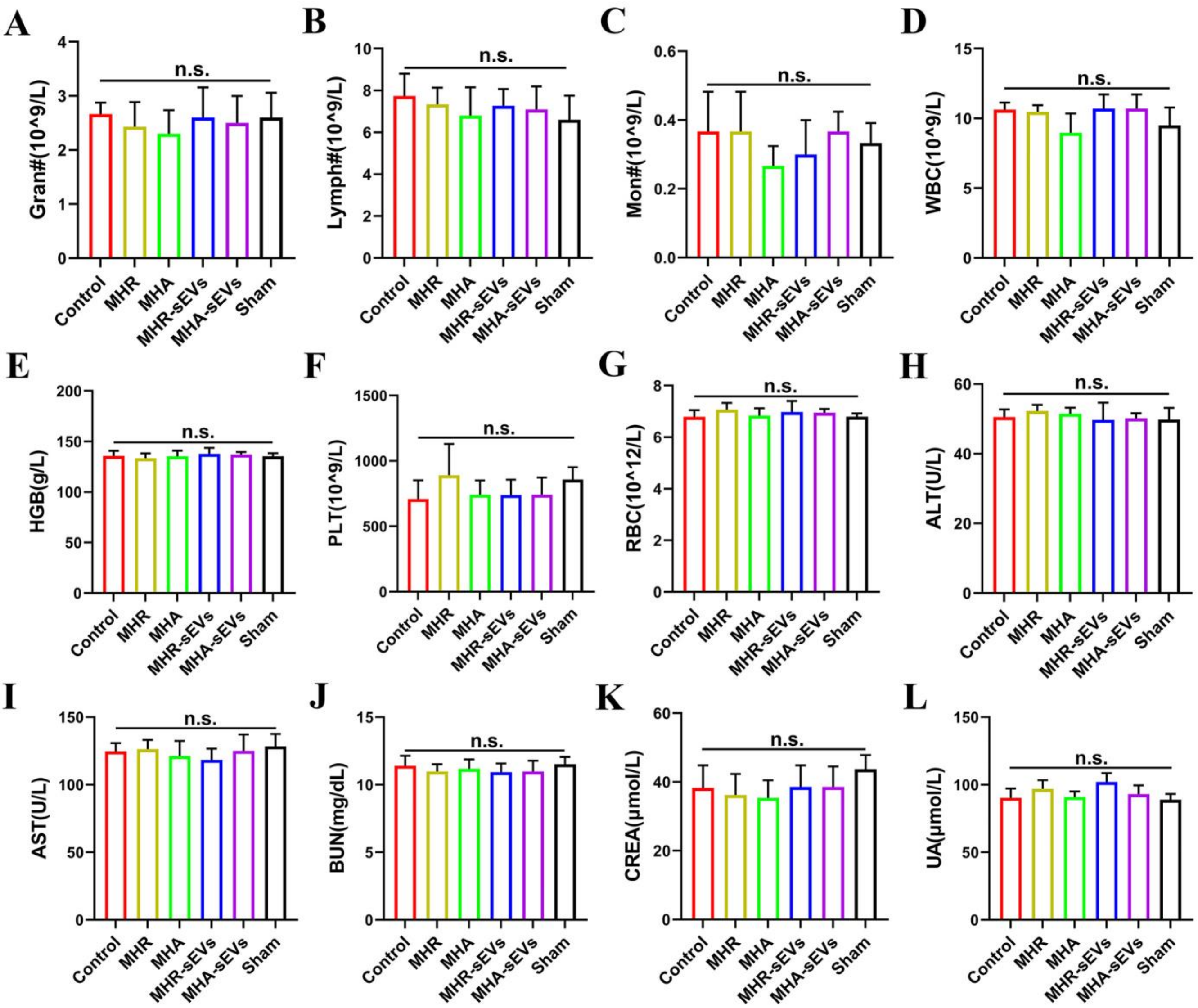

Figure S13. Results of routine blood test and liver and kidney function tests performed for rats at 8 weeks postoperation.

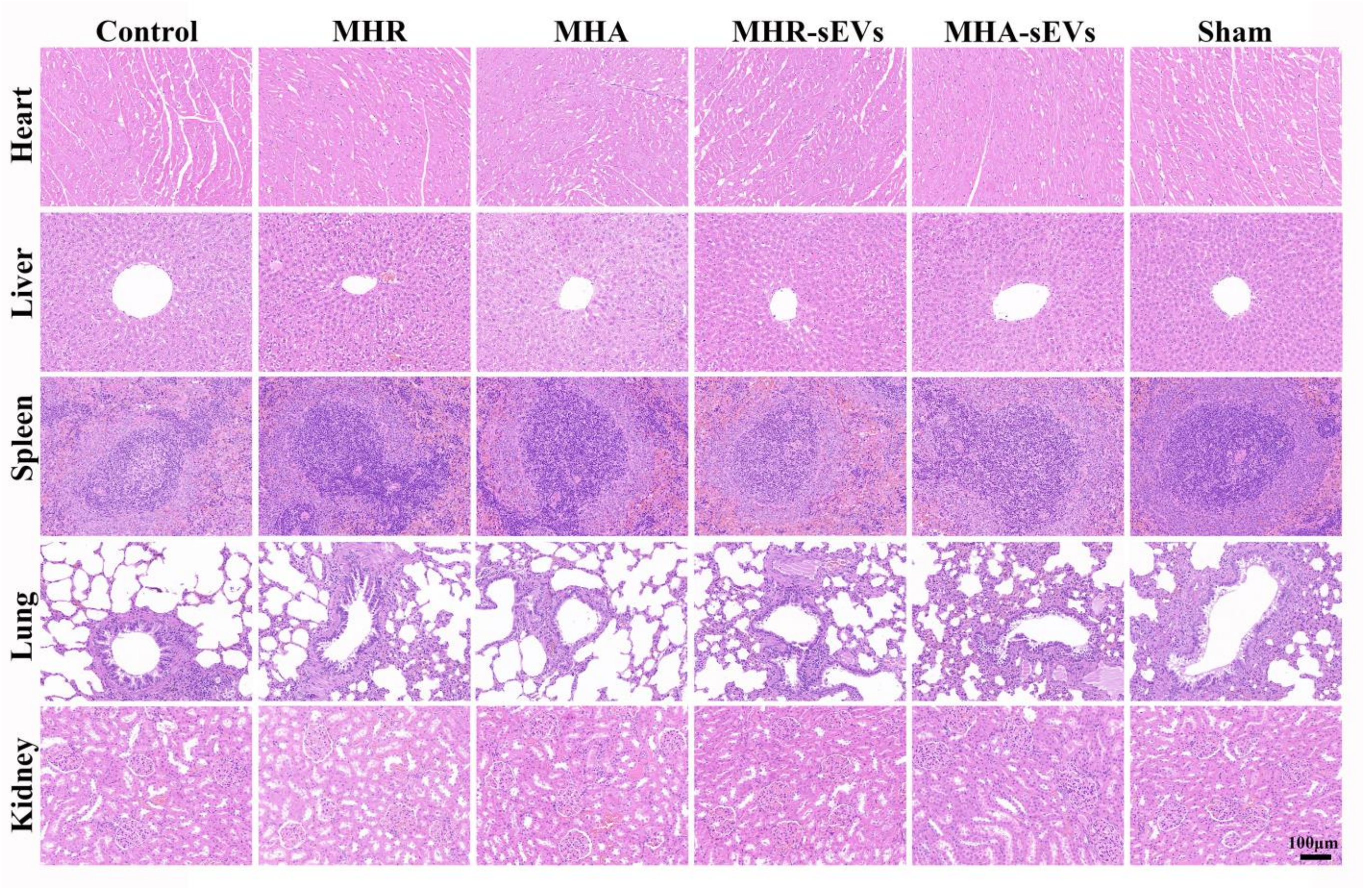

Figure S14. Representative images of H&E staining of major organs (heart, liver, spleen, lungs, and kidneys) explanted from rats at 8 weeks postoperation.

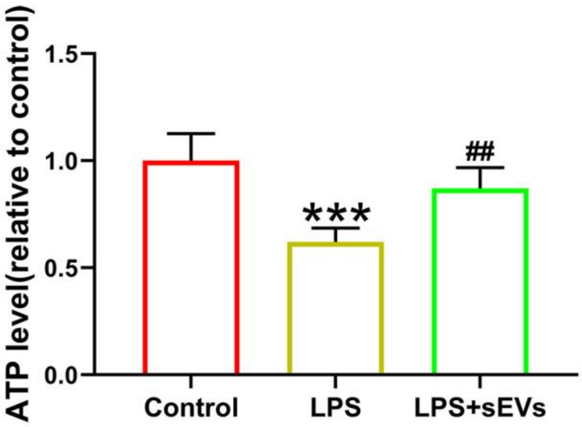

Figure S15. ATP level in Mφ measured by ATP Assay Kit. $^{***}p < 0.001$, when the data were compared with control group. $^{\#\#}p < 0.01$, when the data were compared with LPS group.

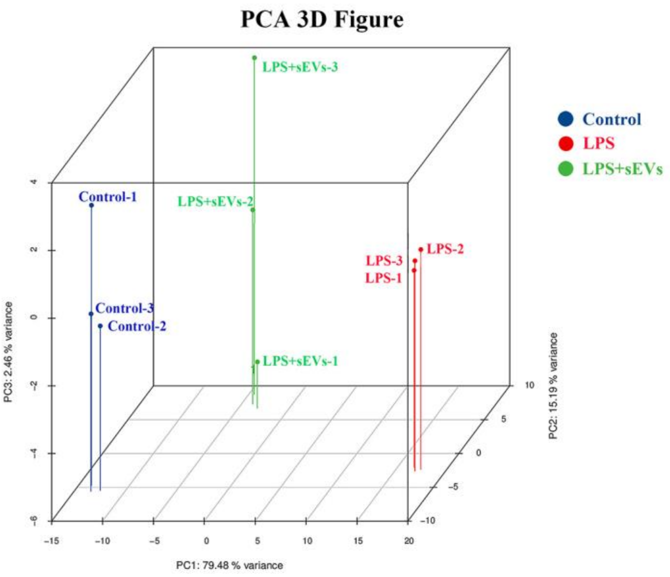

Figure S16. Principal component analysis (PCA) of groups after RNA sequencing.

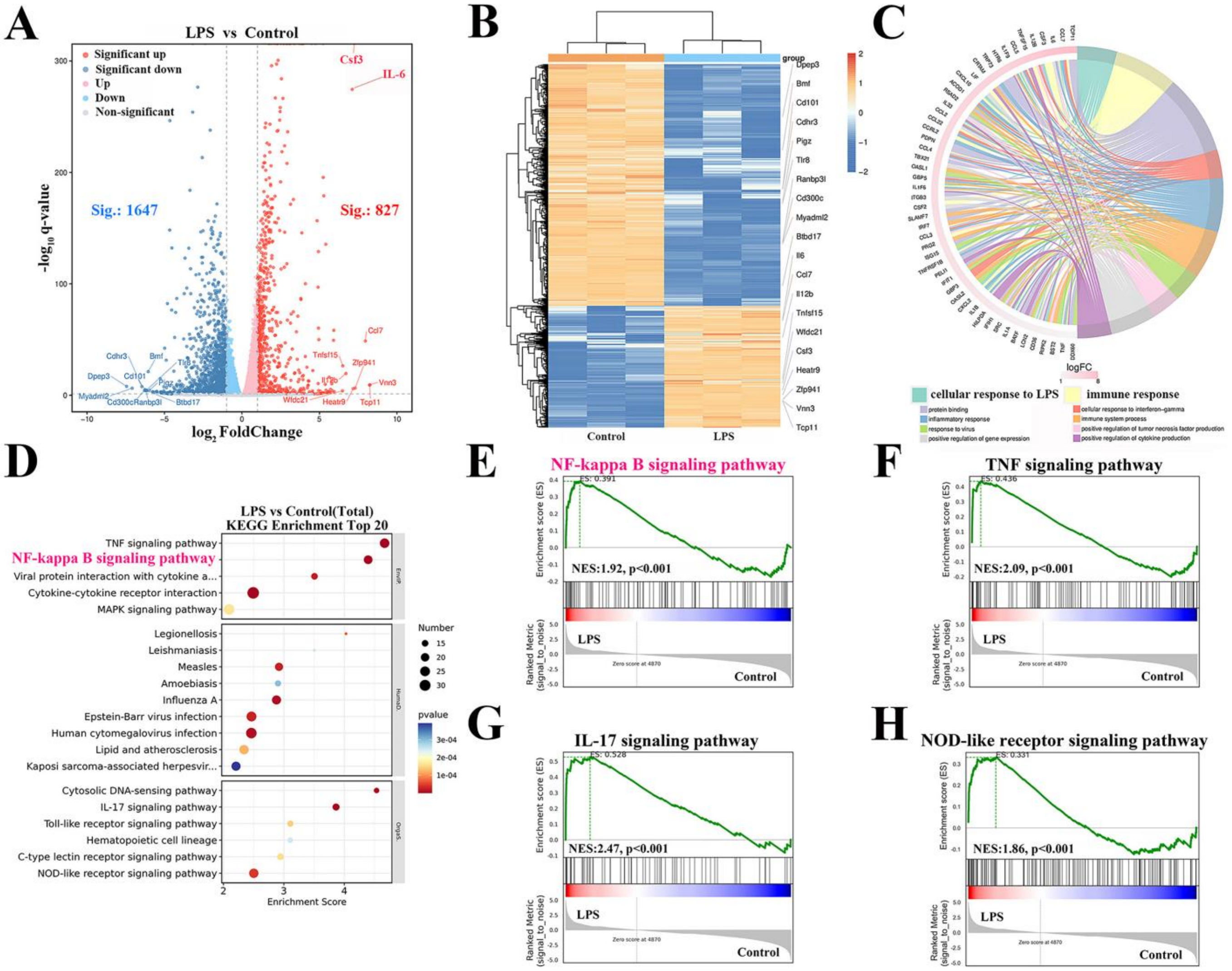

Figure S17. Transcriptome sequencing analysis of M0 and M1 Mφ. A, B) Volcano map and heat map of differentially expressed genes between M1 and M0 Mφ. C, D) GO and KEGG enrichment analysis of differentially expressed genes and pathways. E-H) GSEA analysis of NF-κB, TNF, IL-17, and NOD-like receptor signaling pathways.

Table S1. Primary antibodies for flow cytometry, immunohistochemistry, immunofluorescence, and western blot.

Name	Company	Cat#	Name	Company	Cat#
APC-CD34	Biolegend	378605	COL II	Merck	CP18
APC-CD29	Biolegend	303007	COL III	Servicebio	GB111629
PE-CD44	BD	561858	Scx	Santa Cruz	sc-518082
APC-CD90	BD	561971	Anti-tnmd	Abcam	ab203676
FITC-CD105	BD	561443	CD16/CD32	BD	553141
HSP70	Abcam	ab181606	PE-CCR7	Ebioscience	12-1971-82
Calnexin	Abcam	ab133615	FITC-CD206	Biolegend	141704
TSG101	Abcam	ab125011	INOS	Abcam	ab178945
CD9	Abcam	ab263019	Arginase-1	CST	93668T
CD68	Santa Cruz	sc-20060	β-actin	Proteintech	81115-1-RR
CD86	Proteintech	13395-1-AP	NF-κb p65	CST	8242
CD206	Acam	ab64693	p-NF-κb p65	CST	3033
TNF-α	Servicebio	GB11188	IκBα	Servicebio	GB111509
IL-10	Servicebio	GB11108	p-IκBα	Abcam	ab133462
CD31	Servicebio	GB11063-2	OCN	Proteintech	23418-1-AP
α-SMA	Servicebio	GB13044	VEGFA	Proteintech	66828-1-Ig
Runx2	Proteintech	20700-1-AP	CD31	Proteintech	11265-1-AP
BMP2	Servicebio	GB12252	ALP	Affinity Biosciences	DF6225
COL I	Proteintech	66761-1-Ig			

Abbreviation: Cell Signaling Technology (CST); BD Biosciences (BD).

Table S2. Secondary antibodies for immunohistochemistry, immunofluorescence, and western blot.

Name	Company	Cat#
HRP-conjugated Affinipure Goat Anti-Rabbit IgG(H+L)	Proteintech	SA00001-2
HRP-conjugated Goat Anti-Rabbit IgG H&L	Servicebio	GB23303
HRP-conjugated Goat Anti-Mouse IgG (H+L)	Servicebio	GB23301
FITC-conjugated Goat Anti-Rabbit IgG (H+L)	Servicebio	GB22303
FITC-conjugated Goat Anti-Mouse IgG (H+L)	Servicebio	GB22301
Cy3-conjugated Goat Anti-Rabbit IgG (H+L)	Servicebio	GB21303
FITC-AffiniPure Goat Anti-Rabbit IgG(H+L)	Yeasen	33107ES60
Cy3-AffiniPure Goat Anti-Rabbit IgG(H+L)	Yeasen	33108ES60
Cy3-AffiniPure Goat Anti-Mouse IgG(H+L)	Yeasen	33208ES60

Table S3. Primer sequences used for RT-qPCR.

**Mouse**		
	Forward	Reverse
GAPDH	CCTCGTCCCGTAGACAAAATG	TGAGGTCAATGAAGGGGTCGT
IL-1β	TGCCACCTTTTGACAGTGATG	CATCTCGGAGCCTGTAGTGC
TNF-α	TGGAACTGGCAGAAGAGGCAC	AGGGTCTGGGCCATAGAACTGA
IL-6	TTCTTGGGACTGATGCTGGTG	CACAACTCTTTTCTCATTTCCACGA
Arg-1	CATATCTGCCAAAGACATCGTG	GACATCAAAGCTCAGGTGAATC
IL-1ra	TGTGCCTGTCTTGTGCCAAGTC	GCCTTTCTCAGAGCGGATGAAG
IL-10	TTACCTGGTAGAAGTGATGCCC	GACACCTTGGTCTTGGAGCTTA
**Human**		
GAPDH	GGAAGCTTGTCATCAATGGAAATC	TGATGACCCTTTTGGCTCCC
COL I	CCCCTGGAAAGAATGGAGATGA	CATCCAAACCACTGAAACCTCTG
ALP	TCCTGTTGACACCCCAAACC	CACATGCCCATGCAACACTT
Runx2	GTGGACGAGGCAAGAGTTTCA	TCTGTCTGTGCCTTCTGGGTT
OCN	TCACACTCCTCGCCCTATTG	CTCCTGAAAGCCGATGTGGT
OPN	CGAAGTTTTCACTCCAGTTGTCC	AGGTGATGTCCTCGTCTGTAGC

Table S4. The Modified Tendon Maturing score system for supraspinatus tendon repair.

**Items**	**1**	**2**	**3**	**4**
Cellularity	Marked	Moderate	Mild	Minimal
Percentage of cells resembling tenocytes	< 25%	25%-50%	50%-75%	>75%
Percentage of cells in the parallel direction	< 25%	25%-50%	50%-75%	>75%
Percentage of large diameter fibers characteristic of mature tendon fibers	< 25%	25%-50%	50%-75%	>75%
Percentage of fibers oriented parallel	< 25%	25%-50%	50%-75%	>75%
Vascularity	Marked	Moderate	Mild	Minimal
Reconstruction of tendon-to-bone interface	C(+)I(-)	C(+)I(+) F(-)	C(+)I(+) F(+)T(-)	C(+)I(+) F(+)T(+)

C, continuity; I, ingrowth; F, fibrocartilage; T, tidemark.

References:

[1] K. T. Suh, S. W. Kim, H. L. Roh, M. S. Youn, J. S. Jung, *Clin Orthop Relat Res* **2005**, 220.

[2] D. He, H. Li, *Advanced Functional Materials* **2020**, 30, 2004709.

[3] Z. Ma, W. Song, D. He, X. Zhang, Y. He, H. Li, *Advanced Functional Materials* **2022**, 32, 2113380.

[4] W. Song, Z. Ma, C. Wang, H. Li, Y. He, *J Mater Chem B* **2019**, 7, 6564.

[5] C. M. Hettrich, S. A. Rodeo, J. A. Hannafin, J. Ehteshami, B. E. Shubin Stein, *J Shoulder Elbow Surg* **2011**, 20, 688.

[6] J. Ide, K. Kikukawa, J. Hirose, K. Iyama, H. Sakamoto, H. Mizuta, *J Shoulder Elbow Surg* **2009**, 18, 288.

[7] L. Kong, Z. Wu, H. Zhao, H. Cui, J. Shen, J. Chang, H. Li, Y. He, *ACS Appl Mater Interfaces* **2018**, 10, 30103.

[8] R. Yonemitsu, T. Tokunaga, C. Shukunami, K. Ideo, H. Arimura, T. Karasugi, E. Nakamura, J. Ide, Y. Hiraki, H. Mizuta, *Am J Sports Med* **2019**, 47, 1701.
